# An antisense oligomer conjugate with unpredicted bactericidal activity against *Fusobacterium nucleatum*

**DOI:** 10.1128/mbio.00524-25

**Published:** 2025-04-29

**Authors:** Valentina Cosi, Jakob Jung, Linda Popella, Falk Ponath, Chandradhish Ghosh, Lars Barquist, Jörg Vogel

**Affiliations:** 1 Helmholtz Institute for RNA-based Infection Research (HIRI), Helmholtz Centre for Infection Research (HZI) 557383, Würzburg, Germany; 2 RNA Biology Group, Institute for Molecular Infection Biology (IMIB), University of Würzburg RNA Biology Group 9190, Würzburg, Germany; 3 Cluster for Nucleic Acid Therapeutics Munich (CNATM), Munich, Germany; 4 Department of Biology, University of Toronto 177385, Mississauga, Ontario, Canada; Georgia Institute of Technology, Atlanta, Georgia, USA

**Keywords:** *Fusobacterium nucleatum*, cell-penetrating peptide, peptide nucleic acid, morpholino, antisense antibiotic, envelope stress

## Abstract

Fusobacteria are commensal members of the oral microbiome that can spread from their primary niche and colonize distal sites in the human body. Their enrichment in colorectal and breast cancer tissue has been associated with tumor growth, metastasis, and chemotherapeutic resistance. The use of non-selective antibiotics to remove fusobacteria impairs tumor progression, but prolonged application causes side effects, such as gastrointestinal problems and dysbiosis. Species-specific antisense antibiotics based on peptide nucleic acid (PNA) have shown efficacy in many gram-negative species, suggesting that antisense PNAs may also enable a tailored depletion of fusobacteria. Here, we have investigated the antibacterial potential of cell-penetrating peptide (CPP)-PNA conjugates targeting the mRNA of putative essential genes in *Fusobacterium nucleatum*. Unexpectedly, we observed no growth inhibition with any of the target-specific PNAs but identified a non-targeting control CPP-PNA [FUS79, (RXR)_4_XB-GACATAATTGT] as a potent growth inhibitor of *F. nucleatum*. Our data suggest that the CPP and specific sequence features of FUS79 are responsible for its activity, rather than an antisense effect. Interestingly, FUS79 also inhibits the growth of five additional fusobacterial strains but not of *F. nucleatum* subsp. *vincentii* (FNV). RNA-seq analysis indicates that FUS79 induces a membrane stress response in a vulnerable *F. nucleatum* strain but not in FNV. Collectively, our attempt at developing antisense antibiotics for fusobacteria discovers a potent growth inhibitor, whose bactericidal effect appears independent of target-specific mRNA inhibition.

**IMPORTANCE:**

Enrichment of *F. nucleatum* at cancer sites is associated with increased tumor growth and metastasis. Antibiotic treatment to remove the bacteria was shown to change the course of cancer progression. Here, we explore first steps to establish peptide nucleic acids (PNAs) as specific antisense antibiotics, thereby laying the foundation for further development of antisense technology in fusobacteria. Although the CPP-PNA FUS79 was initially designed as a control, we observed that the compound was bactericidal for specific fusobacterial strains. Our results suggest that FUS79 might be able to selectively deplete fusobacterial strains from bacterial communities, offering a new perspective on fusobacterial removal at the tumor site.

## INTRODUCTION

The human oral microbiome is composed of more than 700 bacterial species from seven different phyla, including the phylum Fusobacteriota (1). Fusobacteria are gram-negative non-motile anaerobic bacteria found in the oral cavity and throat (2). They use their elongated shape and numerous adhesins to connect early and late colonizers of the oral niche (3–5), effectively functioning as a central bridging organism (6–8). One member of this phylum, *Fusobacterium nucleatum*, has received attention for its implication in medical conditions such as oral infections, arthritis, adverse pregnancy outcomes, and most importantly cancer (9). *F. nucleatum* can disseminate throughout the body via the gastrointestinal or hematogenous route and colonize secondary sites (10, 11). Enrichment of *F. nucleatum* at tumor sites is associated with increased cell proliferation, metastasis, inhibition of immune responses, and resistance to chemotherapy (12–14). Antibiotic treatment has been shown to counteract *F. nucleatum*-associated tumor growth (15, 16), but sustained administration of broad-spectrum antibiotics runs the risk of severe side effects such as a systemic inflammatory response and disease reoccurrence (17). This motivates the development of *Fusobacterium*-specific antimicrobial agents for selective removal of this oncomicrobe.

Antisense oligomers (ASOs) have great potential for the development of programmable species-specific antibiotics (18–21). These “asobiotics” are typically designed to base pair with the translation initiation region (TIR) of mRNAs encoding an essential gene in the species of interest. Through antisense sequestration of the TIR, ASOs prevent the synthesis of the targeted protein, resulting in bacterial growth inhibition. Two ASO modalities have been popular for asobiotics design: peptide nucleic acid (PNA) and the phosphorodiamidate morpholino oligomer (PMO). Both are neutral in charge and resistant to cellular nucleases and proteases (22). PNA, the more widely used of the two, is a synthetic DNA analog composed of a peptide-like backbone and natural nucleobases (23). Antisense PNAs composed of 9–12 nucleobases possess a strong binding affinity for DNA and RNA (24) and have been shown to effectively inhibit the translation of target transcripts in *Escherichia coli* or *Salmonella enterica* (24, 25). However, neither PNA nor PMO can passively cross the bacterial envelope (26, 27) and require a carrier for cell entry. The most common approach to facilitate PNA uptake is conjugation to cell-penetrating peptides (CPPs) (28), which are relatively easy to synthesize or modify (29). CPPs have been shown to deliver PNAs into various gram-negative (30) as well as gram-positive (31) bacteria. PNAs conjugated to the polymyxin-inspired peptide (KFF)_3_K or the arginine-rich peptide (RXR)_4_XB have demonstrated potent antimicrobial activity against the gram-negative pathogen *S. enterica* with a typical minimum inhibitory concentration (MIC) in the lower micromolar range (32). PNAs have been extensively used against aerobic gram-negative bacteria (33, 34). In addition, there is one report of their use against *Porphyromonas gingivalis* (35)*,* an obligate anaerobic gram-negative bacterium, with which *F. nucleatum* interacts in the oral cavity. However, PNA-mediated growth inhibition of fusobacteria remained to be tested.

Here, we have attempted to use PNAs as programmable antisense antibiotics to kill fusobacteria. We establish that the CPP (RXR)_4_XB readily enters *F. nucleatum*. However, we found that ASOs conjugated to (RXR)_4_XB targeting mRNAs of essential genes do not inhibit the growth of fusobacteria in culture. Surprisingly, a non-targeting (RXR)_4_XB-PNA conjugate (FUS79) displays potent antimicrobial activity against five different fusobacterial strains. This effect seems to be caused by the combination of the (RXR)_4_XB peptide and certain sequence elements of the conjugated ASO, rather than antisense inhibition of an off-target mRNA. Global RNA-seq analysis reveals that FUS79 elicits a membrane stress response and activates the σE regulon in sensitive fusobacteria but not in resistant fusobacterial species. In summary, our results represent a first step toward the design of ASO therapeutics against *F. nucleatum* and tell a cautionary tale arguing that appropriate controls are needed in the development of CPP-ASOs as antimicrobial agents.

## RESULTS

### Cell-penetrating peptides (KFF)_3_K and (RXR)_4_XB enter *F. nucleatum*

To explore CPPs as potential PNA delivery agents in *F. nucleatum*, we coupled the fluorophore TAMRA to four different CPPs to investigate CPP-mediated uptake into the bacterial cytoplasm, a method previously applied in *E. coli* (36). Using the clinical isolate *F. nucleatum* subsp. *nucleatum* ATCC 23726 (FNN23), we determined the uptake efficiency of the CPPs (KFF)_3_K, (RXR)_4_XB, Seq471, and Seq2373 coupled to TAMRA (Table S1). (KFF)_3_K and (RXR)_4_XB are known to deliver charge-neutral ASOs in numerous gram-negative bacteria (19). Seq471 (WLRRIKAWLRRIKALNRQLGVAA) has a high transport efficiency into HeLa cells without notable cytotoxicity, making it an interesting candidate to target intracellular *F. nucleatum* (37). Seq2373 (AHKLKKPKIVRLIKFLLKAWK) was designed in-house and showed good preliminary uptake efficiency for FNN23 in a flow cytometry screen. Since many CPPs possess an inherent antibacterial activity against gram-negative bacteria (38, 39), we first determined the MIC of the CPP-TAMRA conjugates. Seq471 and Seq2373 displayed an MIC of 10 µM, whereas (KFF)_3_K and (RXR)_4_XB showed an MIC of 20 µM and >40 µM, respectively (Fig. S1A). We therefore tested CPP-TAMRA uptake at 5 µM to avoid toxicity.

**Fig 1:**
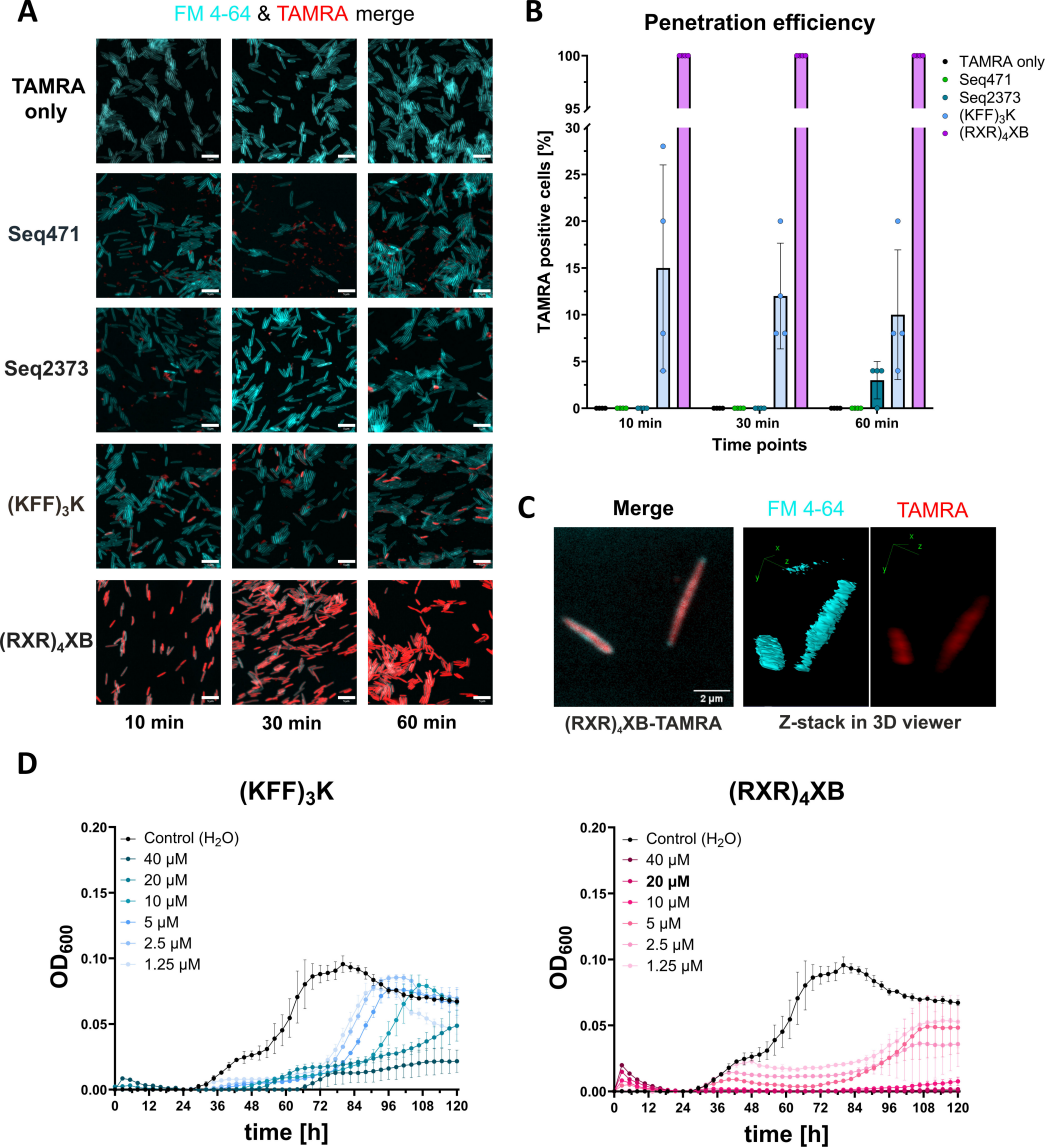
CPP (RXR)_4_XB is a potent delivery tool for *F. nucleatum*. (**A**) Representative CLSM images for TAMRA-only control and TAMRA-labeled CPPs at 10, 30, and 60 min post-treatment. FNN23 was incubated with FM 4–64 to stain the cell membrane, which is shown in cyan. Scale bar, 5 µm. (**B**) Quantification of TAMRA-positive cells for each CPP at selected time points. A total of 100 cells from four different images per condition were selected via bright field, and cytoplasmic TAMRA signals were counted. Each dot represents the percentage of TAMRA positive cells from one image. The mean of four images is shown. Error bars indicate the standard deviation of four images. (**C**) Z-stack of (RXR)_4_XB-TAMRA treated FNN23. IMAGE-J 3D viewer was used to represent z-stacks. (**D**) MIC of unconjugated CPPs using FNN23 in MHB at 1 × 10^5^ CFU/mL input. Growth curves are depicted as OD_600_ over time for three replicates from different overnight (o.n.) cultures, and error bars indicate standard deviation of three experiments. MIC value is indicated in bold.

Using confocal laser scanning microscopy (CLSM), we observed that the uptake of CPPs in FNN23 varies greatly (Fig. 1A). (RXR)_4_XB has strong and stable penetration efficiency with 100% TAMRA-positive cells from 10 min onward. (KFF)_3_K showed ~15% uptake efficiency with a slight decrease over time. Seq471 and Seq2373 showed minimal-to-no translocation with 0%–4% TAMRA-positive cells (Fig. 1B). To confirm that our lead CPP (RXR)_4_XB delivers the fluorophore into the bacteria, we used z-stack recordings to detect (RXR)_4_XB-coupled TAMRA in the cytosol of FNN23 and not in the membrane, the latter judged by membrane staining with FM 4–64 (Fig. 1C).

Since TAMRA conjugation can alter the cytotoxicity of CPPs (40), we determined the MIC of unconjugated (KFF)_3_K and (RXR)_4_XB. The MIC of (KFF)_3_K was >40 µM (Fig. 1D), whereas (RXR)_4_XB showed an MIC of 20 µM (Fig. 1D). Since both CPPs exhibited antibacterial activity at higher concentrations, we selected 10 µM as maximal initial concentration for the following experiments.

### Design of CPP-PNA conjugates to inhibit protein synthesis of fusobacterial essential genes

**Fig 2:**
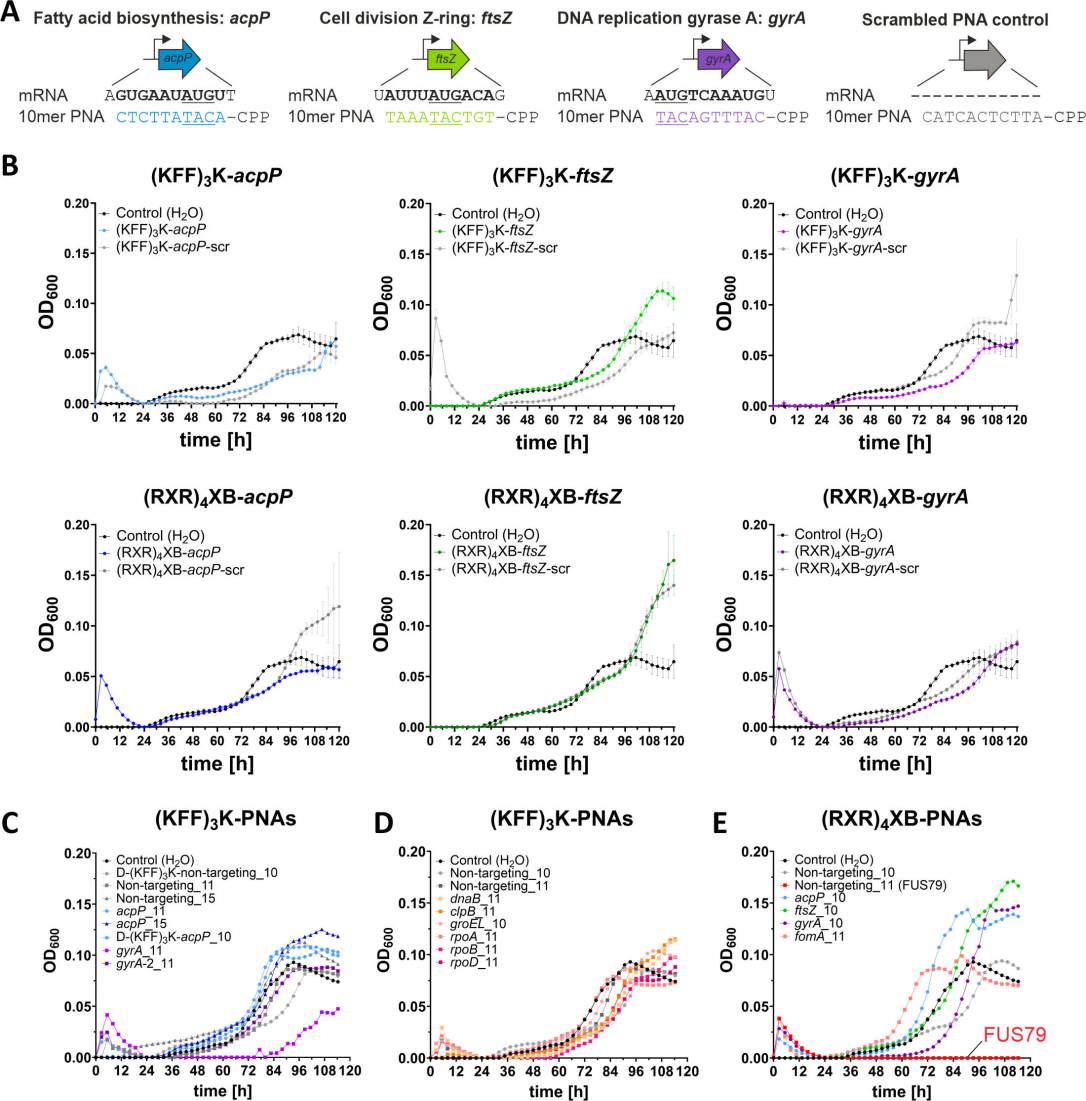
FNN23 growth is not inhibited by mRNA targeting CPP-PNAs, but by a non-targeting (RXR)_4_XB-PNA control. (**A**) Overview of PNA design for three gene targets in FNN23. 10-mer PNAs were designed with sequence complementarity along the translational start codon (“AUG” underlined) of the target mRNAs depicted in bold for *acpP* (blue), *ftsZ* (green), and *gyrA* (purple). For each PNA, the sequence was scrambled as control. An example of a respective scrambled control is shown for the *acpP* sequence (gray). (**B**) Growth inhibition assays of FNN23 using 10 µM CPP-PNAs. Upper row shows (KFF)_3_K-PNAs. On-target PNAs are complementary to *acpP* (light blue), *ftsZ* (light green), and *gyrA* (lilac). Lower row shows (RXR)_4_XB-PNAs. On-target PNAs are complementary to *acpP* (dark blue), *ftsZ* (dark green), and *gyrA* (purple). Corresponding scrambled controls are depicted in gray. All growth curves are depicted as OD_600_ over time for three replicates from different o.n. cultures; error bars indicate the standard deviation of three experiments. (**C**) Growth inhibition of FNN23 with (KFF)_3_K-PNA alternatives for *acpP*, *ftsZ,* and *gyrA*. Non-targeting (KFF)_3_K-PNA controls are shown in gray. D-form (KFF)_3_K CPPs are indicated, respectively. (**D**) Growth inhibition assays of FNN23 using (KFF)_3_K-PNAs. Each color represents a different gene. Non-targeting (KFF)_3_K-PNA controls are shown in gray. (**E**) Growth inhibition of FNN23 with (RXR)_4_XB-PNAs targeting alternative sites of *acpP*, *ftsZ,* and *gyrA* or (RXR)_4_XB-PNA targeting *fomA*. Each color represents a different gene. Non-targeting (RXR)_4_XB-PNA 10-mer control is shown in gray. Non-targeting (RXR)_4_XB-PNA 11-mer control (FUS79) is shown in red. All growth curves were performed in MHB using 1 × 10^5^ CFU/mL and 10 µM CPP-PNAs. Growth is depicted as OD_600_ reading over time.

To identify potential mRNA targets for inhibiting *F. nucleatum* growth, we initially selected three genes assumed to be essential based on inference from other bacterial species (25, 31, 41, 42): *acpP* (C4N14_04130), which encodes acyl carrier protein; *ftsZ* (C4N14_10675), which encodes the Z-ring protein that is important for cell division; and *gyrA* (C4N14_02325), which encodes the gyrase A subunit important for DNA replication (Fig. 2A). For each of these three genes, we designed 10-mer PNAs complementary to the TIR using the previously published FNN23 annotation and the web tool MASON (43–45). To account for unspecific effects, each PNA sequence was scrambled and tested together with the respective targeting PNA as a control.

### Treatment of *F. nucleatum* with CPP-PNAs delays but does not block growth

To test CPP-PNA conjugates for growth inhibition, we diluted mid-exponential phase cultures of FNN23 to 1 × 10^5^ colony forming units (CFUs) per milliliter in Mueller-Hinton Broth (MHB) and incubated the diluted bacteria with 10 µM of either (KFF)_3_K- or (RXR)_4_XB- conjugated PNAs targeting *acpP*, *ftsZ* or *gyrA*. Growth was monitored over time by OD_600_ measurements, with growth inhibition defined as no visible growth (OD_600_ <0.01) after 120 h. Unexpectedly, we did not observe growth inhibition from any CPP-PNA (Fig. 2B). Although some PNAs lead to growth delay, the effect did not seem to be due to target-specific translation inhibition because the respective scrambled controls showed comparable effects (Fig. 2B). Since *ftsZ* inhibition via CRISPRi was reported to prevent FNN23 colony formation on agar plates, but not in liquid culture (46), we tested if the (RXR)_4_XB-PNA targeting *ftsZ* had an effect on CFU numbers. However, we could not observe a CFU reduction after CPP-PNA treatment (Fig. S1B). Based on these findings, we conclude that CPP-PNAs targeting fusobacterial mRNAs are not effective in antisense inhibition.

Next, we explored additional CPP-PNA variations (Table S2) based on (KFF)_3_K-PNAs, since the unconjugated CPP was less toxic to FNN23 compared with (RXR)_4_XB (Fig. 1D). Since PNA length affects efficacy (25, 47), we also tested 11mer and 15-mer PNAs. In addition, we chose a different 10-mer PNA sequence for *gyrA*, shifting the binding site downstream to test if this target window would be more efficient (−1 to +10 relative to AUG start codon; referred to as *gyrA*_2). In addition, we included a D-isomeric form of the lysine in the (KFF)_3_K peptide to avoid putative proteolytic cleavage (30, 48, 49). We used a single non-targeting (KFF)_3_K-PNA control for each PNA with similar length and nucleobase composition. These controls had no predicted binding to any TIR of an annotated FNN23 gene. Of these (KFF)_3_K-PNAs, only the 11-mer *gyrA*-2 PNA caused a substantial growth delay of ~40 h (Fig. 2C). Higher concentrations of this CPP-PNA did not lead to further growth retardation, and CFU spot assays showed a similar effect compared with the non-targeting control (Fig. S1C and D). This suggests that the observed inhibition is most likely mediated by an unspecific effect caused by the CPP-PNA conjugate and not by *gyrA* inhibition.

We then selected eight additional target genes and designed (KFF)_3_K-conjugated 10-mer and 11-mer PNAs (Table S2) targeting mRNAs encoding proteins involved in DNA transcription (*dnaB*, *rpoA*, *rpoB,* and *rpoD*) or stress chaperones (*clpB* and *groEL*) because some of these targets inhibited growth in uropathogenic *E. coli* (25), *Listeria monocytogenes* (50), and *P. gingivalis* (35). None of these eight additional PNAs inhibited fusobacterial growth (Fig. 2D).

### Screening of additional (RXR)_4_XB-PNAs leads to the discovery of FUS79

Since (RXR)_4_XB showed better uptake efficiency in the initial TAMRA screen compared with (KFF)_3_K, we also tested (RXR)_4_XB-conjugated PNAs targeting alternative sequences. Specifically, we designed PNAs targeting sequences around the TIR of *acpP*, *ftsZ,* or *gyrA* as well as an 11-mer PNA targeting *fomA,* a very abundant outer membrane protein of fusobacteria (43, 51). As controls, we included non-targeting 10-mer and 11-mer (RXR)_4_XB-PNAs to account for unspecific effects. None of the on-target PNAs impaired growth (Fig. 2E). However, the non-targeting (RXR)_4_XB-PNA 11-mer control (i.e., (RXR)_4_XB-GACATAATTGT, named FUS79 from here on) completely inhibited growth of FNN23 (Fig. 2E, shown in red). This was unexpected, as this ASO sequence was specifically designed to lack binding sites within TIRs of FNN23, and no other (RXR)_4_XB-PNA conjugate had shown growth inhibition at similar concentrations.

### FUS79 is bactericidal against *F. nucleatum*

**Fig 3:**
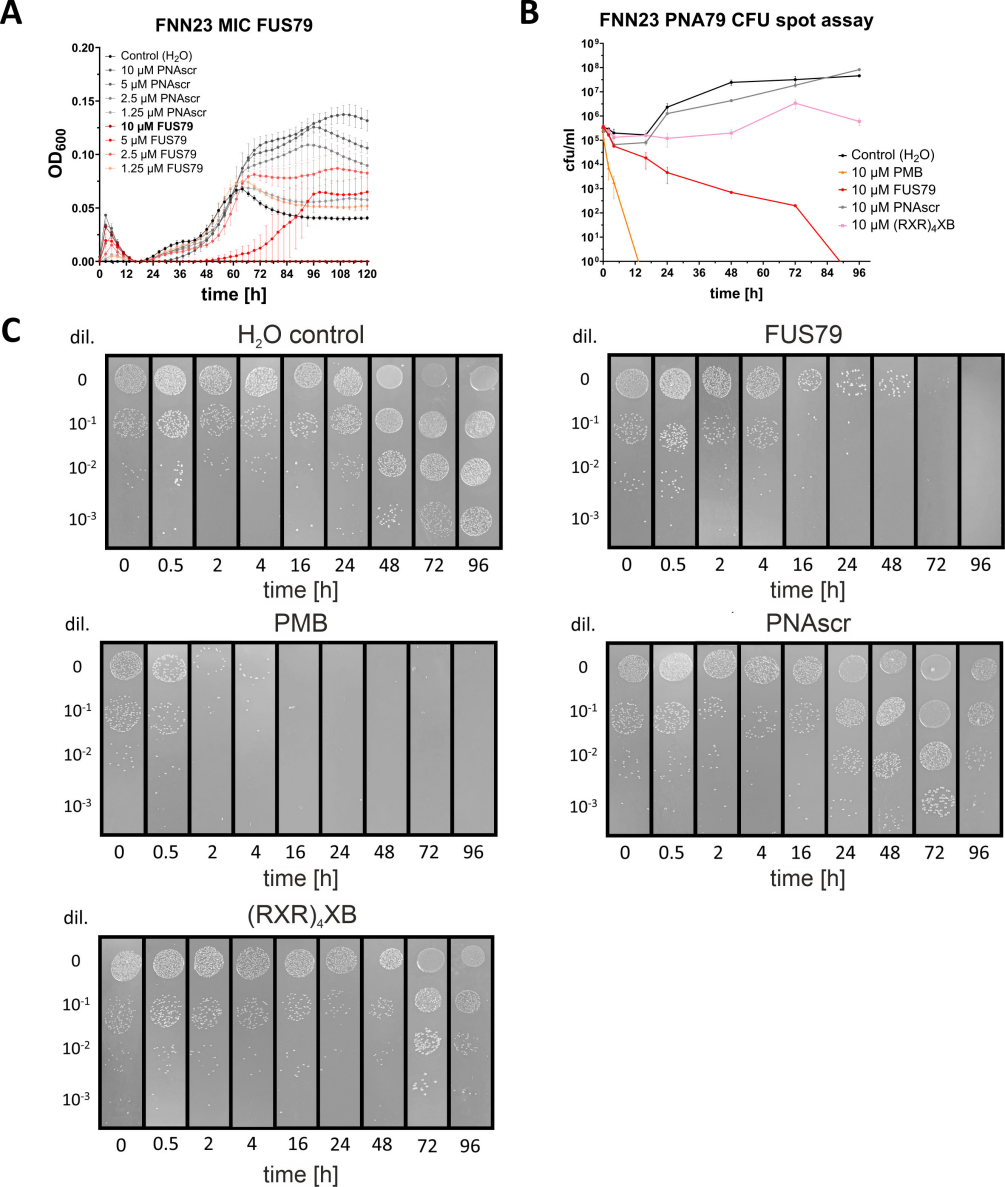
FUS79 is bactericidal against FNN23 at 10 µM. (**A**) Growth kinetics of FNN23 incubated with FUS79 in MHB at 1 × 10^5^ CFU/mL input. The growth curves are depicted as OD_600_ over time for three replicates from different o.n. cultures; error bars indicate the standard deviation of three experiments. MIC value is indicated in bold. (**B**) Determination of bactericidal kinetics for H_2_O as a negative control, polymyxin B (PMB) as a positive control, 10 µM unconjugated (RXR)_4_XB, the corresponding scrambled control (PNAscr), or FUS79. At selected time points post-treatment, 100 µL of each sample were collected and diluted 1:10 in 1× PBS to enumerate the number of viable cells (CFU/mL) via spotting on BHI agar plates. After 3 days, CFUs were counted for three technical replicates and quantified. Error bars represent the standard deviation of three experiments. (**C**) Exemplary images out of three replicates showing CFUs on BHI plates after 3 days of incubation from H_2_O control, FUS79, PMB, PNAscr or (RXR)_4_XB.

To determine the MIC of FUS79, we performed a growth inhibition assay with a dilution series of this PNA. FUS79 leads to complete growth inhibition of FNN23 with an MIC of 10 µM, whereas a scrambled FUS79 sequence (referred to from here on as PNAscr) had no impact on growth (Fig. 3A). To exclude a potential batch effect, we reordered FUS79 from the original vendor and also initiated an in-house synthesis. Both batches confirmed the FUS79-mediated growth inhibition (Fig. S2A and B).

To explore if the observed growth inhibition was due to bacteriostatic or bactericidal effects, we incubated FNN23 with 10 µM of FUS79 and performed CFU spot assays to quantify bacterial viability. Incubation with FUS79 reduced the CFU count from 24 h onward, with no visible CFUs detected on the agar plate after 96 h (Fig. 3B and C). Treatment of FNN23 with PNAscr or unconjugated (RXR)_4_XB had no bactericidal effect. Polymyxin B (PMB), known to have strong bactericidal activity, fully diminished CFUs after 96 h (Fig. 3C). Taken together, our results show that FUS79 completely inhibits the growth of FNN23 at 10 µM.

### FUS79 inhibits the growth of various fusobacterial strains but not *F. nucleatum* ssp. *vincentii*

**Fig 4:**
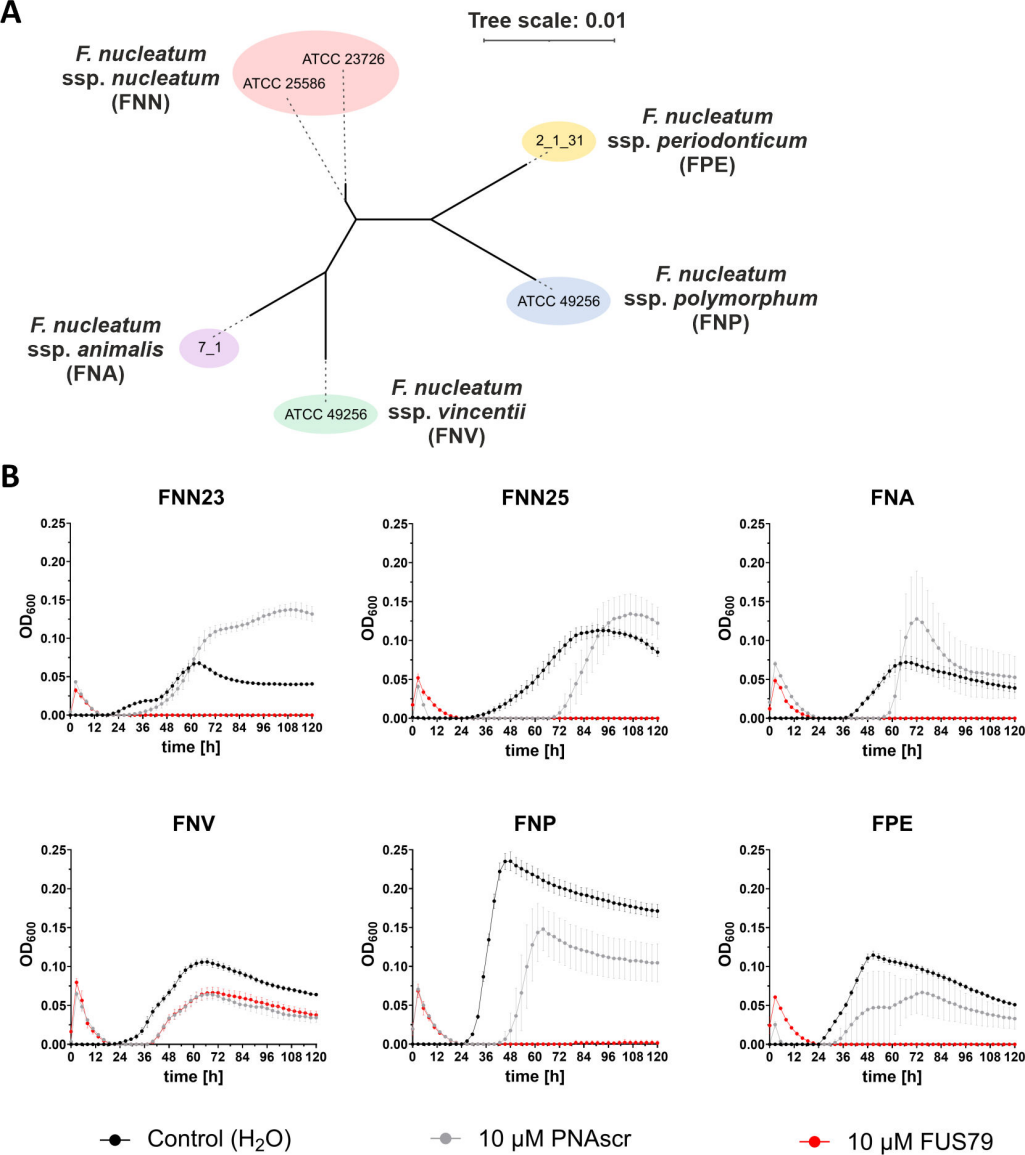
FUS79 inhibits the growth of several fusobacterial species except FNV. (**A**) Phylogenetic tree of F. *nucleatum* strains and FPE was generated using the respective 16S rRNA sequences. Multiple sequence alignment was performed with MAFFT, and a maximum-likelihood phylogenetic tree was then inferred from the aligned 16S rRNA sequences. Branch lengths indicate the expected number of substitutions per site. The scale bar corresponds to 0.01 substitutions per site, that is, one substitution per 100 bases. (**B**) Growth kinetics of FNN23, FNN25, FNA, FNV, FNP, and FPE in MHB at 1 × 10^5^ CFU/mL input incubated with 10 µM FUS79 (red) or the corresponding PNAscr (gray). Growth curves are depicted as OD_600_ over time for three replicates from different o.n. cultures; error bars indicate the standard deviation of three experiments.

To better understand the intriguing bactericidal activity of FUS79, we tested whether the observed bactericidal effect was exclusive to FNN23. We performed growth inhibition assays with five other fusobacterial strains associated with human physiology and pathology (52): *F. nucleatum* subsp. *nucleatum* ATCC 25586 (FNN25), *F. nucleatum* subsp. *animalis* 7_1 (FNA), *F. nucleatum* subsp. *vincentii* ATCC 49256 (FNV), *F. nucleatum* subsp. *polymorphum* ATCC 10953 (FNP), and *F. periodonticum* 2_1_31 (FPE) (Fig. 4A). These strains differ in their growth rate. FNN23 and FNA display a long lag phase and show regrowth in MHB after 24–40 h with a maximum OD_600_ value below 0.1. In contrast, FNN25, FNV, FNP, and FPE show steady regrowth around 24 h and reach higher OD_600_ values (Fig. 4B).

Remarkably, FUS79 inhibited the growth of all strains, except FNV (Fig. 4B). Although FNV is phylogenetically closer to FNN23 than, for example, FNP or FPE (Fig. 4A), the latter strains are both susceptible to FUS79. When treating the different strains with the PNAscr, we observed growth retardation compared with the water control, which might be attributed to the antibacterial activity of (RXR)_4_XB itself. These results indicate that the bactericidal effect of FUS79 is not limited to FNN23 but is common among different fusobacterial strains.

### FUS79 does not inhibit the growth of several other gram-negative bacteria

**Fig 5:**
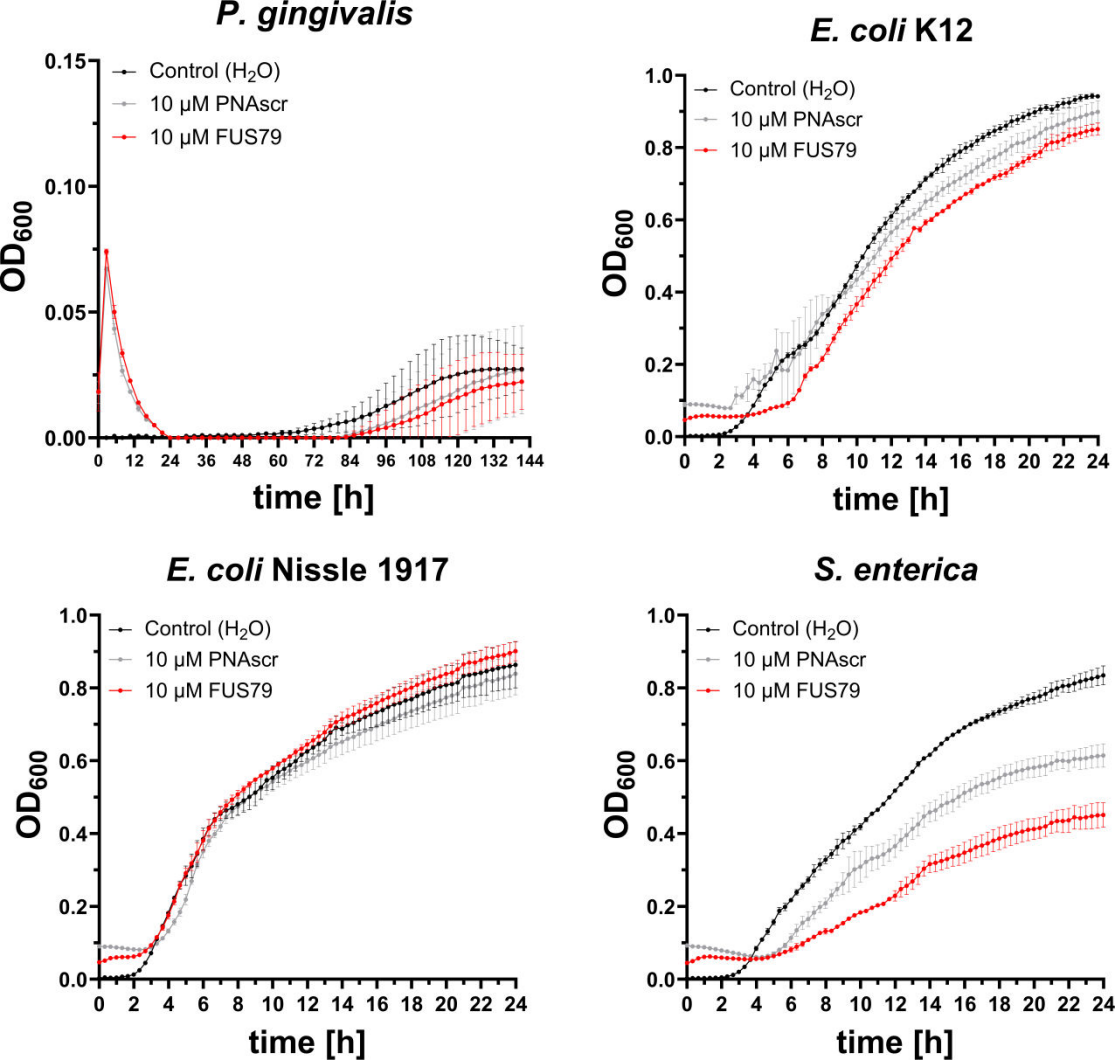
FUS79 does not specifically inhibit the growth of other gram-negative bacteria. Growth kinetics of *P. gingivalis*, *E. coli* K12, *E. coli* Nissle 1917, and *S. enterica* incubated with 10 µM FUS79 or the respective PNAscr control in MHB using 1 × 10^5^ CFU/mL input. For anaerobic growth, OD_600_ was measured for 144 h, whereas for aerobic growth, data were collected for 24 h. Growth curves are depicted as OD_600_ over time for three replicates from different o.n. cultures; error bars indicate the standard deviation of three experiments.

To investigate if FUS79 shows similar bactericidal effects against other bacterial species, we tested its effect on *P. gingivalis*, a gram-negative anaerobic bacterium that shares the oral niche and co-aggregates with *F. nucleatum* (53). We also included *E. coli* K12 and *E. coli* Nissle 1917 as representative gut commensals (54). Incubation with FUS79 showed no growth inhibition for these species, similar to the PNAscr control (Fig. 5). These data suggest that FUS79 might specifically inhibit certain fusobacterial strains. Furthermore, we tested the effect of FUS79 against *Salmonella enterica* serovar Typhimurium SL1344. We had shown previously that incubation of *S. enterica* with 10 µM (RXR)_4_XB-PNAs causes a growth defect (32), which we confirm here (Fig. 5 bottom right). This suggests that (RXR)_4_XB-PNAs generally impair the growth of *S. enterica* and that FUS79 has no specific bactericidal effect.

### The growth inhibitory capacity of FUS79 does not seem to be mediated by off-target regulation

The nucleobase sequence of FUS79 (GACATAATTGT) has no predicted full antisense match within any TIR in FNN23 or FNN25, and no full-length complementary sequences in the genomes of FNA, FNP, and FPE. However, we found 19 potential FUS79 TIR off-targets in FNN23 if we allowed one or two mismatches. We reasoned that those potential off-targets are worth investigating since terminal double mismatches in a 10-mer PNA do not necessarily abrogate growth-inhibitory activity (45). Since FUS79 showed growth inhibition in five fusobacterial strains, but not in FNV, we searched for mismatch TIR off-targets present in the five vulnerable strains but absent in the resistant FNV. We found one off-target that fits these criteria: FUS79 is complementary to the TIR of *trpB* mRNA (tryptophan synthase subunit beta; C4N14_04955 in FNN23) with two terminal mismatches, that is, GACATAATTGT. We tested if a fully complementary PNA sequence targeting *trpB* inhibits the growth of the most vulnerable strain FNN23. Treatment with 10 µM (RXR)_4_XB-*trpB* did not result in growth impairment, arguing against a potential translational inhibition of *trpB* as the mode of action of FUS79 (Fig. S2C).

### Antimicrobial activity of FUS79 is mediated by (RXR)_4_XB and distinct PNA sequence elements

**Fig 6:**
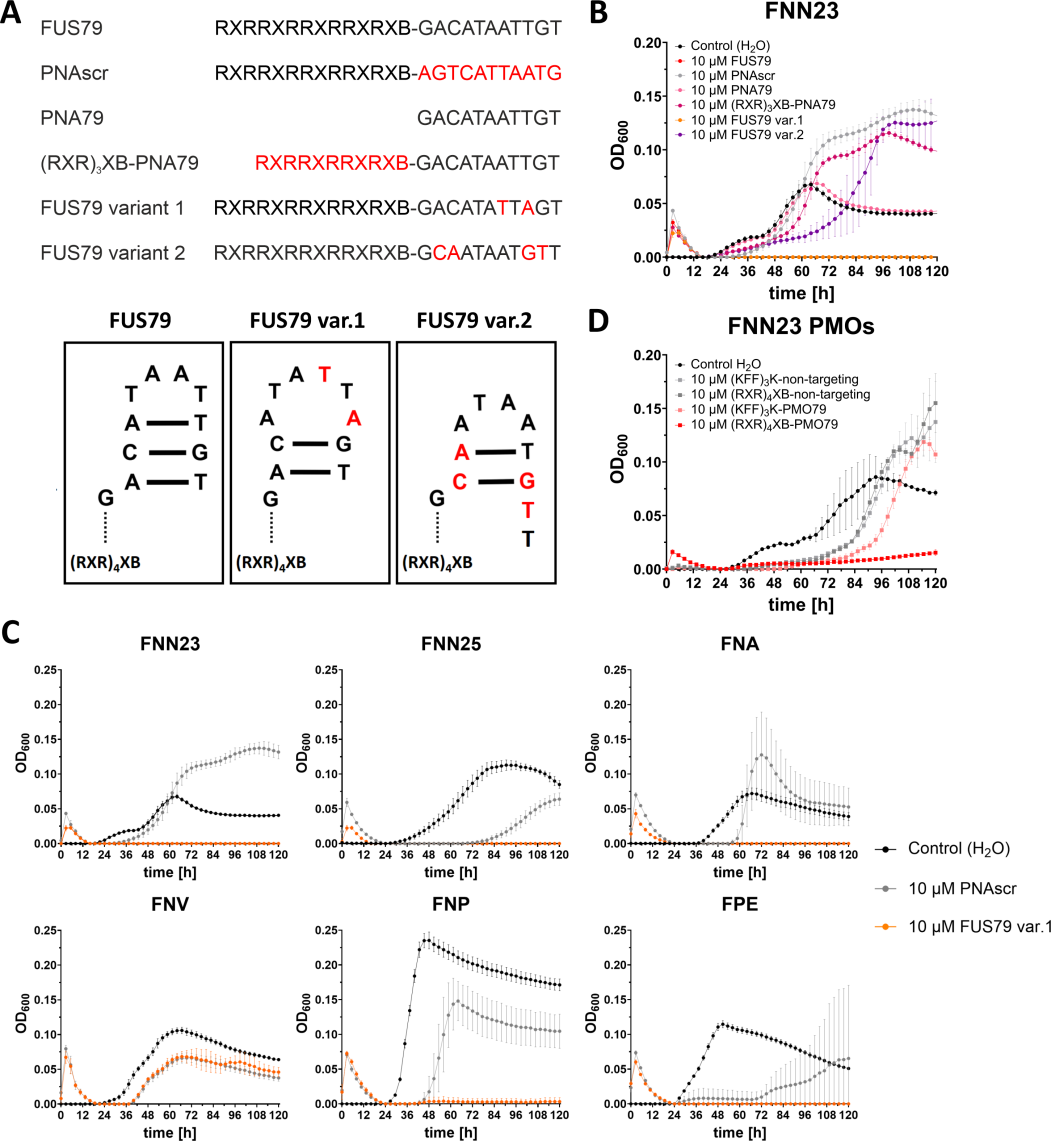
Bactericidal effect of FUS79 is dependent on (RXR)_4_XB and certain sequence elements of PNA79, but independent of ASO modality. (**A**) (Top) List of investigated FUS79 variants. Changes with respect to the original conjugate are depicted in red. (Bottom) Schematic representation of the putative hairpin structure of PNA79. Nucleobase exchanges, shown in red for variant 1 and variant 2, including the presumed changes in structure. (**B**) Growth kinetics of FNN23 incubated with CPP-PNAs or PNAs at 10 µM concentration. The growth curve of FUS79 (red) is nearly identical to variant 1 (orange) and mostly overlaps with the orange line. (**C**) Growth kinetics of FNN23, FNN25, FNA, FNV, FNP, and FPE incubated with 10 µM FUS79 variant 1 (orange) and corresponding PNAscr (gray). (**D**) Growth kinetics of FNN23 incubated with 10 µM of (KFF)_3_K- or (RXR)_4_XB-conjugated PMO79 are shown in red. Water control is depicted in black, respective non-targeting PMO controls are in gray. All growth curves are shown as OD_600_ over time for three replicates from different o.n. cultures with 1 × 10^5^ CFU/mL input in MHB; error bars indicate the standard deviation of three experiments.

Since FUS79 did not seem to inhibit *F. nucleatum* growth through TIR targeting, we further investigated the potential reasons for the observed toxicity in the five vulnerable strains. To this end, we tested FUS79 without the (RXR)_4_XB module (namely PNA79), with a shortened (RXR)_4_XB module (namely (RXR)_3_XB), coupled to (KFF)_3_K instead of (RXR)_4_XB (Fig. S3A), and two sequence variants of the original FUS79 (Fig. 6A). These PNA sequence variants contain nucleobase substitutions expected to disrupt a predicted 3 bp hairpin structure of FUS79 (Fig. 6A). Although single-stranded PNAs are presumed to form compact structures due to their flexible backbone and hydrophobicity, they have also been shown to form hairpins under certain conditions (55). After performing growth inhibition assays with FNN23, we observed that only the original FUS79 as well as sequence variant 1 conjugated to full-length (RXR)_4_XB inhibited the growth of FNN23 at 10 µM (Fig. 6B). Conjugating PNA79 to (KFF)_3_K did not lead to growth inhibition (Fig. S3A). When testing these compounds in the other fusobacterial strains, only FUS79 and sequence variant 1 were effective in strains susceptible to FUS79 but not in the resistant strain FNV, highlighting a common mode of action between the original FUS79 and variant 1 (Fig. 6C). Therefore, we conclude that intact (RXR)_4_XB together with specific parts of the FUS79 sequence (GACATAWTWGT) appear to be essential for the bactericidal effect.

We also tested different experimental conditions, that is, higher bacterial inoculum density and alternative bacterial culture media. These experiments showed that at 10 µM, FUS79 is no longer bactericidal at 10^7^ CFU/mL in MHB (Fig. S3B) and has no effect when tested in peptide-rich media such as brain heart infusion (BHI) or Columbia broth (ColB) (Fig. S3C). This demonstrates the MIC of FUS79 depends on the inoculum size and medium type, which is common in many antibiotics and antimicrobial peptides (56, 57).

To further explore if the antibacterial activity was dependent on the ASO backbone modality, we replaced the PNA moiety with a phosphorodiamidate morpholino oligomer (PMO) while retaining the nucleobase sequence of FUS79. Although antisense-mediated translation inhibition by PMO typically requires a longer sequence compared to PNA (58, 59), we decided to keep the same nucleobase length to directly compare the two conjugates, since the mode of action of FUS79 is unlikely to be mediated by sequence-specific binding. The PMO-ASO was conjugated to either (RXR)_4_XB or (KFF)_3_K as control since (KFF)_3_K-PNA79 had shown no bactericidal effect. At 10 µM concentration, (RXR)_4_XB-PMO79 showed similar growth inhibition as FUS79, although the backbone of PMO is very different from PNA. In contrast, the (RXR)_4_XB-conjugated non-targeting PMO control as well as (KFF)_3_K-PMO79 did not inhibit the growth of FNN23 (Fig. 6D). This result suggests that the bactericidal effect is due to the combination of the ASO nucleobase sequence and (RXR)_4_XB.

### RNA-seq analysis of a sensitive and resistant *F. nucleatum* strain upon FUS79 treatment

**Fig 7:**
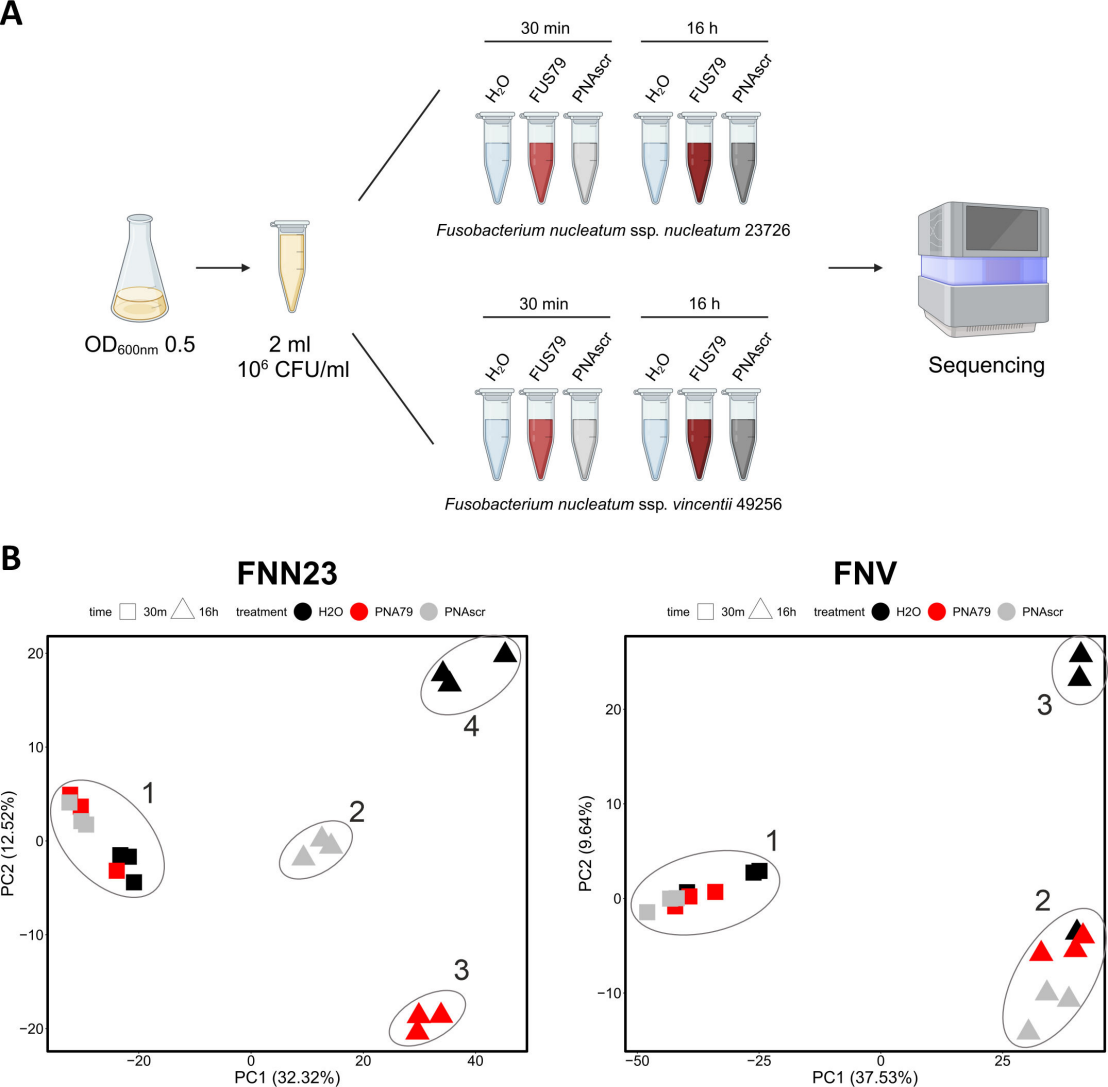
PCA plot reveals distinct clusters for FUS79-treated sensitive FNN23 versus resistant FNV. (**A**) Experimental workflow showing the different samples subjected to RNA-seq. Image has been created with BioRender.com. (**B**) Principal component analysis (PCA) of 18 samples for each fusobacterial strain after TMM normalization. Clusters were inserted manually, grouping closely positioned samples.

To investigate the mechanism of FUS79, we employed RNA-seq to measure transcriptomic changes associated with treatment in a sensitive fusobacterial strain. We chose FNN23, which was the strain most susceptible to FUS79, as well as FNV, which was resistant. We treated both strains with FUS79, the corresponding PNAscr, or water as control and took RNA-seq data at 30 min to monitor early transcriptomic changes prior to the onset of growth inhibition and at 16 h after the onset of the bactericidal activity (Fig. 7A).

To obtain an overview of the data, we evaluated the results using principal component analysis (PCA; Fig. 7B; Fig. S4). When comparing PC1 vs. PC2 (Fig. 7B) at 30 min, there were no large differences between samples for either strain. In contrast, at 16 h post-treatment, we observed distinct patterns of clustering for FNN23 and FNV, as might be expected, given the different sensitivity of these strains to FUS79. In FNN23, the water control, FUS79, and the PNAscr formed three distinct clusters, indicating disparate responses to these three treatment conditions. In contrast, for FNV samples, we observed only two clusters: one containing two of the water-treated control samples and a second containing both FUS79- and PNAscr-treated samples as well as a single water-treated control, which was removed from further analysis. PC1 vs. PC3 (Fig. S4A) as well as PC2 vs. PC3 (Fig. S4B) showed a similar clustering and did not further help to separate the samples. These results indicate that a 16-h FUS79 treatment elicits a distinct response from the PNAscr in strain FNN23, which is sensitive to the ASO, but not in the resistant strain FNV.

To investigate general transcriptomic responses of fusobacteria to CPP-PNA treatment, we compared FUS79 as well as PNAscr-treated samples to the water control (Supplementary data SD1). We mapped the reads using the *F. nucleatum* subsp. *nucleatum* ATCC 23726 annotation file (NZ_CP028109.1) with custom annotation and the *F. nucleatum* subsp. *vincentii* 3_1_36A2 annotation file (NZ_ CP003700.1). We observed that while FUS79 and PNAscr do not induce significant changes (log_2_ fold change < −1.5 or > 1.5 with an adjusted false discovery rate of ≤0.01) after 30 min in FNN23, both induce downregulation of transcripts involved in purine metabolism after 16 h (C4N14_07225-C4N14_07265, Fig. S5). Upregulated transcripts differ strongly between the two CPP-PNA treatment conditions (Fig. S5). For FNV, only *hemC*, which is important for heme biosynthesis, is upregulated upon PNAscr treatment after 30 min. At 16 h post-treatment, both CPP-PNAs downregulated *pckA* and *pdxS*, involved in gluconeogenesis and cofactor biosynthesis, respectively. Furthermore, mRNAs HMPREF0946_RS04730, HMPREF0946_RS03355, and HMPREF0946_RS08775 as well as sRNA FunR47 are downregulated, but there are no commonly upregulated transcripts (Fig. S5). In conclusion, there does not appear to be a common CPP-PNA-induced transcriptomic response in fusobacteria.

### FUS79 induces the σE membrane stress response in the sensitive *F. nucleatum* strain

**Fig 8:**
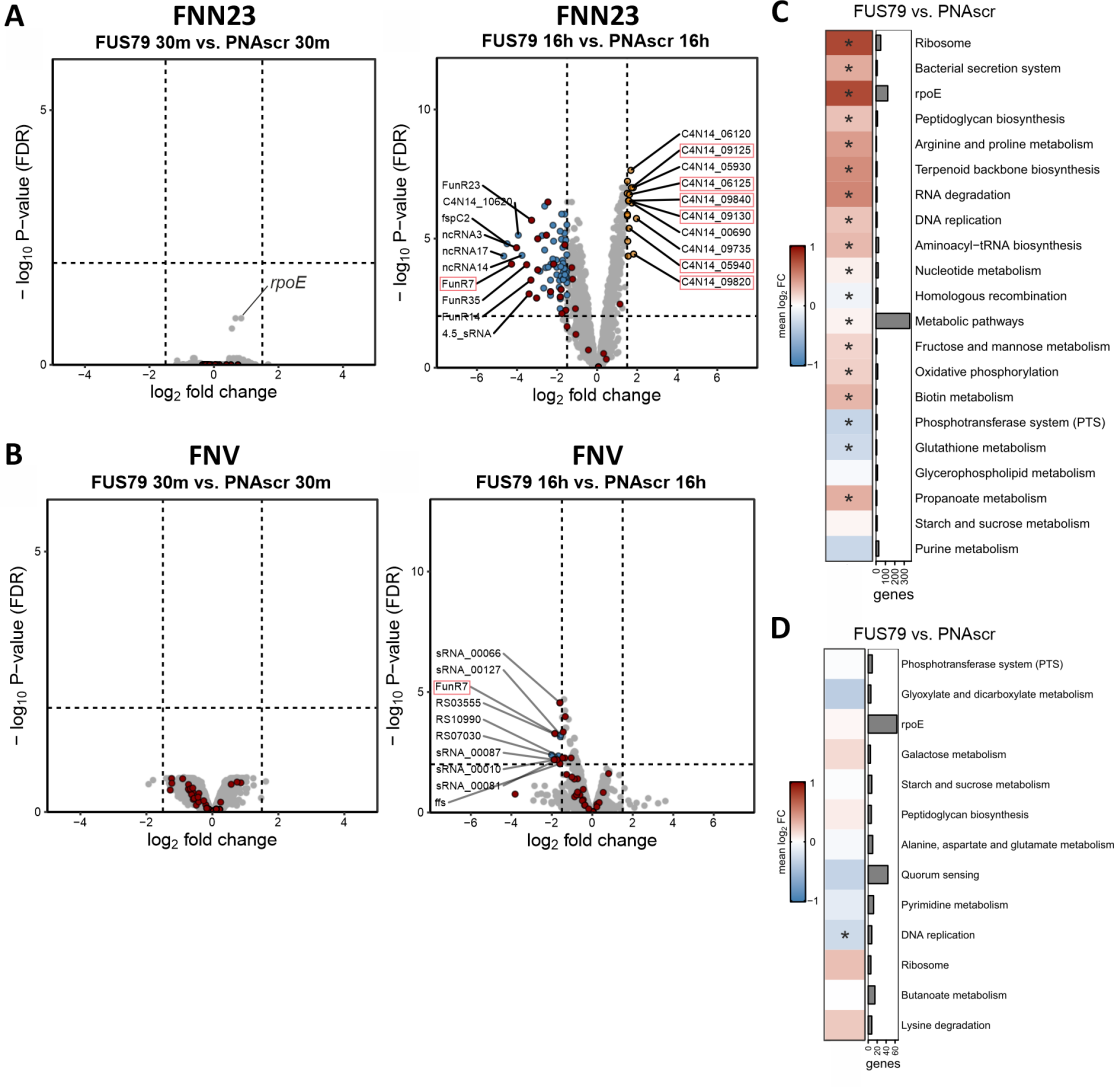
FUS79 treatment induces σE-dependent membrane stress response in sensitive FNN23. (**A, B**) Transcriptomic response of FNN23 (**A**) and FNV (**B**) upon FUS79 or PNAscr treatment. Volcano plots show differential gene expression as –log_10_ false discovery rate (FDR)-adjusted *P* values on the *y*-axis and log_2_ fold change on the *x*-axis. (Left) Thirty minutes 10 µM FUS79 vs. 10 µM PNAscr. (Right) Sixteen hours 10 µM FUS79 vs. 10 µM PNAscr. Significantly differentially expressed transcripts are defined by log_2_ fold changes <−1.5 or >1.5 and an FDR adjusted *P* value <0.01, depicted as dashed lines in the plot. Significantly upregulated transcripts are depicted as orange dots, and significantly downregulated transcripts as blue dots. All sRNAs are colored as red dots. The top 10 differentially expressed transcripts are specified by the indicated locus tag. Gene locus tags that are part of the σE (*rpoE*) regulon are red-boxed. (**C, D**) KEGG pathway enrichment analysis of annotated gene sets and the manually added σE regulon for FNN23 (**C**) or FNV (**D**) after 16 h of treatment. RNA-seq data of FNV and FNN23 was analyzed using annotated KEGG pathways. *, FDR-adjusted *P* value adjusted <0.05. Gene number per pathway is indicated on the right, and all pathways are shown as median log_2_ fold change with the corresponding color shade as indicated in the legend on the left.

To further characterize the distinct transcriptional response to FUS79, we focused on the differences between FUS79 and PNAscr (Fig. 8), although we have made results for all relevant comparisons available (Fig. S5). In keeping with our PCA analysis, we see minimal differences in RNA levels in both FNN23 and FNV after 30-min treatment with either of the two CPP-PNAs (Fig. 8A and B). However, at 16 h following CPP-PNA treatment, we observe pronounced differences between FUS79 and PNAscr in FNN23 samples. Specifically, we found a total of 82 differentially expressed transcripts between FUS79 and PNAscr samples (Fig. 8A). Interestingly, six of the top 10 upregulated transcripts upon FUS79 treatment compared with PNAscr belong to the σE regulon of FNN23 (60) (marked with red boxes in Fig. 8A). The σE regulon plays an important role in the global stress response in gram-negative bacteria and is composed of multiple proteins and small RNAs (sRNAs) involved in the maintenance of envelope integrity (61, 62). Recently, the σE regulon has been studied in *F. nucleatum* showing an oxygen-induced stress response reminiscent of the activated σE response in Proteobacteria (60). Indeed, closer inspection of the transcriptome after 30-min treatment shows that the most highly upregulated gene is σE itself (*rpoE*, C4N14_03400; indicated in Fig. 8A, left panel).

The function of σE was shown to be supported by two sRNAs, namely FoxI and FoxJ, which regulate mRNAs of envelope proteins (44). However, neither FoxI nor FoxJ was differentially expressed in FNN23. Of all the differentially expressed sRNAs, many were downregulated upon FUS79 treatment, but none were upregulated (red dots, Fig. 8A). Of the sRNAs that are part of the σE regulon, only FunR7 was significantly downregulated. Furthermore, the 4.5S signal recognition particle RNA (4.5S RNA), which is generally involved in bacterial protein synthesis as well as in directing the translation of membrane and secretory proteins to the inner membrane (63, 64), is among the top 10 downregulated transcripts. The two strongest downregulated mRNAs are C4N14_10620, a hypothetical protein, and *fspC2*, a small ORF uncharacterized in fusobacteria. Collectively, our RNA-seq analysis shows upregulation of transcripts involved in the membrane stress response of FNN23 upon FUS79 treatment, with particular upregulation of transcripts in the σE regulon.

The resistant strain FNV displays no significantly upregulated transcripts upon FUS79 treatment compared with the PNAscr after 16 h (Fig. 8B). Six of the 10 most downregulated transcripts are sRNAs including FunR7 and the 4.5S RNA (ffs), possibly indicating a partly common response to FUS79 shared between both strains. Apart from the shared downregulation of FunR7 and 4.5S RNA, the transcriptomic response to FUS79 versus PNAscr has no other common aspects between the vulnerable FNN23 and the insensitive FNV strain. In conclusion, we observed that the transcriptomic responses between FNN23 and FNV are distinct, and only the susceptible strain shows upregulation of the σE regulon.

### FUS79 generates general stress responses in FNN23 but not in FNV

To put our differential expression analysis in the context of functional pathways, we performed a gene set enrichment analysis using the KEGG database supplemented with the σE regulon of *F. nucleatum*. We found that upon FUS79 treatment, there was a strong induction of KEGG pathways associated with ribosomes, arginine and proline metabolism, bacterial secretion systems, peptidoglycan synthesis, RNA degradation, and terpenoid backbone synthesis in the vulnerable strain FNN23 (Fig. 8C). Many of these pathways are linked to a general stress response, response to antibiotic treatment, or membrane homeostasis (65–67). As seen above, the σE regulon was strongly upregulated as well. Two pathways associated with the phosphotransferase system and glutathione metabolism were downregulated. These pathways are mainly involved in carbohydrate transport, transcription regulation, stress response, and the regulation of cell division (68–70). In contrast, analysis of the resistant FNV strain revealed no pathways that were significantly upregulated and only the DNA replication pathway to be significantly downregulated (Fig. 8D).

Taken together, our RNA-seq analysis revealed a strong transcriptomic response characterized by the upregulation of the σE regulon, antibiotic stress responses, and membrane homeostasis after 16 h for FUS79 compared with PNAscr in FNN23 but not in the resistant strain FNV.

## DISCUSSION

In our attempt to design species-specific asobiotics targeting *F. nucleatum*, we found that although (RXR)_4_XB was able to efficiently penetrate FNN23, none of the CPP-ASOs that we designed to target putative essential mRNAs inhibited bacterial growth. Instead, we observed an intriguing bactericidal effect of a PNA designed as a non-targeting control against five fusobacterial strains. Below, we propose potential reasons for our inability to establish target-specific antibacterial ASOs in fusobacteria, such as poor cytosolic delivery of CPP-PNA conjugates, limited knowledge of gene essentiality, as well as PNA design. Furthermore, we discuss the potential mechanisms of FUS79 and why FNV might be resistant. Overall, our observations highlight the importance of considering and reporting unanticipated antibacterial activities, which is essential when evaluating CPP-ASOs as tools for precision microbiome editing (20).

### Exogenously delivered ASOs show no sequence-dependent antisense activity in *F. nucleatum*

Several arguments support the idea that PNAs might function effectively in fusobacteria. First, *F. nucleatum* possesses a large number of regulatory sRNAs (44, 60, 71). Inferring from gram-negative model species of bacterial RNA biology (72, 73), the majority of these fusobacterial sRNAs can be assumed to repress or activate target mRNAs by targeting their 5’ end. Indeed, we have already shown that the *F. nucleatum* sRNA FoxI represses *fomA* mRNA by base pairing around the start codon (60). Thus, fusobacterial mRNAs are permissive to antisense modulation. Second, in line with previous reports of arginine-rich peptides as efficient ASO delivery agents in several gram-negative species (74), we demonstrated that fluorophore-labeled (RXR)_4_XB efficiently penetrates the membrane of FNN23. Third, we screened many genes that are essential and shown to be amenable to PNA-mediated inhibition in bacteria such as *E. coli* (25, 75), *S. enterica* (76), *Staphylococcus aureus* (77), *K. pneumoniae* (74)*,* and *Buchnera aphidicola* (78).

Although of the ASO targets we tested, only *ftsZ* is experimentally confirmed to be essential in FNN23 (46), and we expect the other targets to encode essential gene products as well based on observations from other bacteria (79, 80). That said, despite initial transposon screens and the recent establishment of CRISPRi for FNN23 and FPE (46, 79, 81, 82), we lack a complete picture of gene essentiality in fusobacteria. It is also possible that the designed PNA sequences are not efficient enough to induce growth inhibition. The AT-rich genome of fusobacteria makes it difficult to design PNAs with a high melting temperature and low self-complementarity (83, 84), both important factors for ASO efficiency (47, 85). Moreover, the exact binding site on the mRNA target also greatly influences the translational inhibitory capacity of an ASO (86). It might be that we have not yet identified the best target region to inhibit mRNA translation with ASOs in fusobacteria. A sequence tiling screen within TIRs might help identify the most vulnerable binding window to increase ASO efficiency. However, the slow and fastidious growth of fusobacteria complicates the establishment of robust growth assays with a clear separation of ASO-triggered growth inhibition versus normal growth fluctuations.

Although (RXR)_4_XB was able to deliver TAMRA into fusobacteria, it might not effectively deliver the much larger PNAs to the bacterial cytoplasm. Indeed, the delivery efficiency of CPPs was shown to depend greatly on the chemical properties of the cargo (24, 87). It is therefore worth considering testing alternative ASO delivery vehicles for *F. nucleatum*. Gold nanoparticles, vitamin B12, and DNA-tetrahedrons have been reported to facilitate ASO uptake by gram-negative and gram-positive bacteria (84, 88–90). Another promising delivery approach is siderophores because these small Fe(III) chelating molecules produced by bacteria (91) are not cytotoxic. Indeed, siderophores have been employed in a “Trojan horse” strategy to deliver antibiotics to bacteria (92). They have also demonstrated good delivery efficacy of PNAs and PMOs into *E. coli* (93, 94). Although fusobacteria lack confirmed siderophore biosynthesis genes, they might be able to utilize xenosiderophores, that is, iron-chelating compounds produced by other members of the microbiota as shown for *Clostridioides difficile* (95). It remains to be seen which other delivery mechanisms are feasible for ASO delivery into *F. nucleatum*.

Overall, our results highlight that although asobiotics represent a promising antibacterial strategy for multiple gram-negative species, application to non-model bacteria faces challenges, highlighting the need for further studies to systematically investigate ASO delivery and application in non-model bacteria.

### Bactericidal effect of FUS79

We have identified a non-targeting (RXR)_4_XB-PNA (FUS79), which is bactericidal against several fusobacterial strains. It seems unlikely that FUS79 inhibits the growth of sensitive strains via an antisense off-target mechanism. First, FUS79 has no fully complementary sequence within any TIR in FNN23. Second, the 19 potential FNN23 TIR off-targets with one or two terminal mismatches as well as transcriptome-wide off-targets were not significantly deregulated after FUS79 treatment in our RNA-seq experiments. As some bacterial sRNAs were shown to prefer imperfect matches (96), we investigated the transcript levels of the mismatched TIR off-target *trpB* (C4N14_04955). However, also *trpB* did not show significant mRNA downregulation upon FUS79 treatment compared with the PNAscr control (Supplementary data SD1). Third, FUS79 has no fully complementary sequence in any genomic region of FNA, FNP, or FPE. Fourth, the FUS79 sequence variant 1, which has two nucleobase substitutions at non-terminal positions, showed the same antibacterial effect as FUS79, although the resulting mismatches should hamper binding to a FUS79 complementary sequence. Nevertheless, we cannot exclude that mismatched off-targets might lead to translational inhibition without affecting mRNA abundance. In that regard, it was shown that target mRNA depletion is not a universal trait of PNA-mediated translation inhibition (25).

Our RNA-seq profiles of FNN23 bacteria treated with FUS79 show upregulation in transcripts involved in membrane homeostasis. The bactericidal effect of FUS79 might therefore be mediated by membrane damage triggered by the specific CPP and ASO sequence combination. The resistance of FNV could be mediated by lipopolysaccharide (LPS) modifications affecting CPP entry. Based on genomic analysis, the LPS O-antigen of FNV is different compared with the sensitive FNN25. FNV possesses genes necessary for the incorporation of sialic acid, galactopyranose, galacturonate, and colitose into LPS, whereas other *F. nucleatum* subsp. *nucleatum* strains do not (97). Indeed, changes in the LPS composition of *E. coli* were shown to affect the uptake of CPP-PNA conjugates (98). That said, the sensitive FNP also incorporates sialic acid into its O-antigen (99), and FNV (RXR)_4_XB-TAMRA uptake rates are similar to the sensitive FNN23 (Fig. S6).

### FUS79 is a strong inducer of the fusobacterial σE response

We found that FUS79 induces many transcripts involved in membrane biosynthesis and homeostasis in the vulnerable FNN23 strain. KEGG pathway analysis further confirmed that the σE regulon was strongly induced. It is worth noting that we added this gene group manually because recent studies showed that more genes are part of the σE regulon than computationally predicted in the KEGG database (44, 60). An enrichment in this regulon could therefore also result from the high number of individual upregulated transcripts present in multiple pathways and not be strictly σE-specific. Nevertheless, the upregulation of *rpoE* in response to FUS79 after 30 min indicates an induction of the σE stress response. The σE regulon is activated via numerous stressors such as heat shock, osmotic stress, pH stress, oxidative stress, or antibacterial agents (61, 100–102). We cannot link the activation of σE to membrane stress specifically, as the KEGG analysis demonstrated the activation of diverse stress responses. In FNN23, activation was recently shown to be induced upon oxygen exposure rather than membrane stressors such as polymyxin B or lysozyme (60). However, these experiments were conducted in cation- and peptide-rich ColB, where bacteria are generally less sensitive to antimicrobial compounds like CPPs (103, 104), and FUS79 did not retain its bactericidal effect. In contrast, in this study, we used MHB, which might lead to an overall higher sensitivity to membrane-targeting antimicrobials.

We propose that the unique combination of (RXR)_4_XB and the PNA79 sequence might interact with the bacterial envelope and lead to σE regulon activation as well as a membrane stress response with subsequent growth inhibition upon membrane disruption. Further mechanistic studies are needed to determine the precise mode of action of FUS79. The MIC of FUS79 depended on the medium type and inoculum size, as is often described for membrane-acting antimicrobial agents (56, 105, 106). Moreover, a (KFF)_3_K-conjugated PNA79/PMO79 did not impair bacterial growth (Fig. S3A; Fig. 6D), supporting the hypothesis that the cytotoxicity is dependent on (RXR)_4_XB and certain sequence elements. It would be interesting to test if a CPP with a similar uptake efficiency and entry mode as (RXR)_4_XB would display bactericidal activity when coupled to PNA79. This would test whether (RXR)_4_XB is specifically needed for the toxicity of FUS79 or whether it depends on the cellular delivery of the ASO component. In general, the conjugation of a CPP to an ASO changes the overall chemical properties as well as the secondary structure of both components, which might influence the cell-penetrating and target binding efficiency of the conjugate (105, 107, 108). Although the net positive charge of (RXR)_4_XB should be unaffected by conjugation to the charge-neutral PNA or PMO modalities (41, 109), changes in the CPP conformation are more likely. These changes might lead to increased penetration as membrane disruption was shown to be dependent on the CPP structure in gram-negative bacteria (110).

### Outlook

Broad-spectrum antibiotics can deplete *F. nucleatum* from the tumor site, leading to therapeutic benefit, but such treatment would disrupt the patient’s protective microbiota. Species-specific ASOs could offer a solution to this problem. We tried to establish asobiotics against *F. nucleatum* but were not able to achieve antisense-mediated growth inhibition. However, it remains unclear whether the CPP-PNAs effectively reach the fusobacterial cytoplasm and are able to bind their mRNA target. Mass spectrometry of cellular fractions could be a feasible approach to test CPP-PNA localization, but our preliminary experiments were unsuccessful. As discussed above, further systematic analyses are required to explore asobiotics as antimicrobial agents against *F. nucleatum*.

Additional therapeutic alternatives to depleting *F. nucleatum* could be species-specific lytic phages, such as FNU1 (111), narrow-spectrum antibiotics (112), species-specific antimicrobial peptides (113), targeting the bacterium through chemically modified transfer RNA fragments (114), tailored CRISPR antimicrobials (115), or the administration of monoclonal antibodies targeting fusobacterial proteins (116, 117). FUS79 might also be considered a lead for the development of a selective inhibitor while sparing resident microbiota. Interestingly, FUS79 showed bactericidal activity against several fusobacterial strains, with no effect on *P. gingivalis* or *E. coli* strains. However, FUS79 exhibits an unidentified bactericidal mechanism that seemingly operates independently of target inhibition. We have tried coupling FUS79 to fluorophores or biotin, but the conjugations abolished the bactericidal effect, which limits the interpretation of localization with these modified conjugates.

Identifying the molecular mechanism of FUS79 will be essential for its clinical application. The membrane interaction of FUS79 with *F. nucleatum* could be mechanistically investigated using different approaches such as membrane model systems, membrane dyes, or molecular dynamics simulations. Such assays bear challenges, since the envelope structure of *F. nucleatum* is largely unknown, and the exposition to atmospheric oxygen triggers membrane stress response itself (60). However, by leveraging the power of medicinal chemistry with respect to chemical modalities and modifications, it should be possible to further enhance the potency of FUS79 to fully harness its potential as a specific antibacterial agent.

## MATERIALS AND METHODS

### Strains and growth conditions

The following fusobacterial strains were used in this study: *Fusobacterium nucleatum* subsp. *nucleatum* ATCC 23726 (FNN23) acquired from the American Type Culture Collection (ATCC), *F. nucleatum* subsp. *nucleatum* ATCC 25586 (FNN25), *F. nucleatum* subsp. *vincentii* ATCC 49256 (FNV), and *F. nucleatum* subsp. *polymorphum* ATCC 10953 (FNP) acquired from the German Collection of Microorganisms and Cell Culture (DSMZ), *F. nucleatum* subsp. *animalis* 7_1 (FNA) and *F. periodonticum* 2_1_31 (FPE) both received as a kind gift from E. Allen-Vercoe (University of Guelph, Canada). All strains were routinely grown at 37°C in 80:10:10 (N_2_:H_2_:CO_2_) anaerobic conditions on 2% agar supplemented BHI plates (BHI, 1% [wt:vol] dried yeast extract, 1% [vol:vol] 50% sterile-filtered glucose solution, 5  µg/mL of hemin; 1% [vol:vol] fetal bovine serum) from frozen 20% glycerol stocks kept at −80°C. For liquid growth, the strains were cultured in Columbia broth (ColB; BD Difco). Precultures were prepared 24 h before inoculating the working culture at a 1:50 dilution in fresh ColB until OD_600_ 0.5 was reached. Cultures were then diluted in non-cation adjusted Mueller-Hinton Broth (MHB, BD Difco) to the desired CFU/mL number. *P. gingivalis* (DSM 20709) was purchased from DSMZ and grown at 37°C in anaerobic conditions as mentioned above on 2% agar supplemented BHI+ plates (BHI, 1% [wt:vol] dried yeast extract, 1% [vol:vol] 50% sterile filtered glucose solution, 5  µg/ml of hemin; 10% [vol:vol] fetal bovine serum; and 1 µg/mL vitamin K3) for 4 days. For liquid growth, *P. gingivalis* was cultured in BHI+ medium (BHI, BD Difco supplemented with 1% [vol:vol] 50% sterile-filtered glucose solution, 5  µg/mL of hemin; 1% [vol:vol] fetal bovine serum, and 1 µg/mL vitamin K3). Precultures were prepared 24 h before inoculating the working culture at a 1:20 dilution in BHI+ until OD_600_ of 0.5 was reached. All plates, media, buffers, and reagents were brought into the anaerobic chamber the day before usage to ensure full oxygen depletion.

Aerobic bacterial culture was conducted with *E. coli* K12 MG1655 (provided by D. Lee), *E. coli* Nissle 1917, and *S. enterica* serovar Typhimurium SL1344 (provided by D. Bumann, Biocenter Basel, Switzerland). The strains were streaked on lysogeny broth plates, incubated o.n. at 37°C and cultured in non-cation-adjusted MHB with aeration at 37°C and 220 rpm constant shaking.

### Cell-penetrating peptides (CPPs) and peptide nucleic acids (PNAs)

CPPs, PNAs, and CPP-PNA constructs were obtained from Peps4LS GmbH, where all compounds were tested with mass spectrometry and HPLC to assess their quality and quantity. PMOs were purchased from GeneTools, LLC. In-house CPP-PNA synthesis was performed by W. Tegge and B. Kornak at the Helmholtz Centre for Infection Research (HZI, Braunschweig). PNA sequences were designed with the help of MASON (45), and PNA scrambled sequences were verified to have no off-targets in translation initiation regions by manual searches in the whole genome sequence of FNN23 (NZ_CP028109). Comparisons with the other fusobacterial strains were also conducted manually using the genome files NZ_AE009951.2 for FNN25, NZ_CP007062.1 for FNA, NZ_CP003700.1 for FNV, NZ_CM000440.1 for FNP, and NZ_CP028108.1 for FPE. To ensure solubility and correct stock preparation, all compounds were briefly vortexed for three seconds, spun down, heated at 55°C for 5 min, and then again vortexed and centrifuged. Stocks (200 µM) were prepared in water; concentration was determined via Nano-Drop spectrophotometer measurements at *A*_205_ for CPPs or *A*_260_ for PNAs and adjusted if necessary. CPP and PNA stocks were stored at −20°C, low binding tips, as well as low binding tubes (Sarstedt), were used throughout for handling.

### Conjugation of PMOs to CPPs

Peptide-conjugated PMOs were prepared following a previously published protocol, with minor modifications (24). Briefly, 65 nmol of CPP with a terminal lysine azide was dissolved in ~15 µL of water and added to 50 nmol of PMO with a terminal cyclooctyne moiety dissolved in 50 µL in a centrifuge tube. The reaction mixture was shaken at 500 rpm at 25°C overnight. As quality control, RP-HPLC analysis of each peptide-conjugated PMO was performed on a JASCO HPLC system (AS-4050, PU-4180, CO-4060, MD-4010, FP-4025) using a PerfectSil 300 ODS reversed-phase column (C18, 250 × 4.6 mm, 100 Å, 5 µm) from MZ-Analysentechnik GmbH (Mainz, Germany). The peptide-conjugated PMOs were further characterized using MALDI mass spectrometry. The samples were stored as lyophilized powders at −20°C until use.

### Confocal laser scanning microscopy (CLSM) for the investigation of CPP uptake

To investigate the efficiency of different cell-penetrating peptides, we excluded a flow cytometry approach to quantify penetration efficiency because upon cell fixation, CPPs were reported to bind to cell membranes and remain attached even after multiple washes, which would result in a false-positive penetration signal in flow cytometry (118). Thus, we opted for a microscopy-based investigation with lower throughput but discrimination potential between membrane-associated and cytosolic signals. CPPs were coupled to the fluorophore 5(6)-carboxytetramethylrhodamine (TAMRA) by Peps4LS GmbH, and 200 µM stocks were prepared in house. Briefly, a bacterial overnight culture was diluted to 10^8^ CFU/mL in fresh non-cation-adjusted MHB (BD Difco, Thermo Fisher Scientific), incubated with 5 µM CPP-TAMRA at 37°C with 230 rpm in the anaerobic chamber, taken out of the chamber, centrifuged at 4°C for 10 min with 13,000 × *g* to collect the pellet, fixated with 4% (wt:vol) PFA at 4°C for 10 min, stained with the membrane dye N-(3-Triethylammoniopropyl)−4-(6-(4-(diethylamino)phenyl) hexatrienyl)pyridinium dibromide (FM 4–64 Biomol, 1:1,000 diluted in water) at RT for 15 min, washed once with 1× PBS, and 1–2 μL of cell suspension spotted on a 1.5% agar pad. Samples were imaged using ibidi chambers on a Leica SP5 laser scanning confocal microscope (Leica Microsystems) at the corresponding wavelengths. The emission was detected at 685–795 nm for FM 4–64 and 570–620 nm for TAMRA. CLSM images were analyzed using ImageJ. To quantify CPP uptake, 100 cells were chosen out of four images per condition using the bright field image and analyzed for cytosolic TAMRA signal by excluding TAMRA-negative cells and TAMRA signal co-localized with the FM 4–64 signal.

### MIC determination and growth inhibition assays

To determine the MIC value, broth microdilutions were performed according to the standard protocol with some modifications (119). An overnight bacterial culture was diluted in a fresh ColB medium and grown to OD_600_ 0.5 (mid-exponential phase). Fusobacteria were then diluted 1:2,500 or 1:250 in fresh MHB to obtain 1 × 10^5^ CFU/mL or 1 × 10^6^ CFU/mL as indicated. To determine the growth inhibition of *P. gingivalis,* an o.n. culture grown in BHI+ was diluted 1:1,500 in MHB to obtain 1 × 10^5^ CFU/mL. *E. coli* K12, *E. coli* Nissle 1917, and *S. enterica* o.n. cultures grown in MHB were diluted 1:1,800 in fresh MHB to obtain 1 × 10^5^ CFU/mL. In total, 190 µL of diluted bacterial cultures were pipetted into a transparent 96-well plate (Nunc, Thermo Fischer Scientific) together with 10 µL of respective 20× CPP or CPP-PNA stock or water as control. Bacterial growth was monitored by measuring OD_600_ every 20 min with constant shaking in a plate reader (Biotek) under the normal atmospheric environment or positioned in the anaerobic chamber with double orbital shaking every 20 min prior to each measurement with 237 cpm at 37°C for 120 h. The MIC was defined at the lowest concentration in which growth was visibly inhibited (OD_600_, <0.01 for anaerobic cultures and <0.1 for aerobic cultures).

### Determination of bactericidal effect using spotting

To investigate the bactericidal effects of PNAs an o.n. culture of FNN23 grown in ColB was diluted 1:50 in fresh ColB and grown to OD_600_ 0.5. This culture was then diluted 1:2,500 to 1 × 10^5^ CFU/mL in MHB, and 190 µL of the culture was dispensed into a transparent 96-well plate (Nunc, Thermo Fischer Scientific) together with 10 µL of respective 20× PNA stock or water and incubated at 37°C with 237 cpm shaking every 20 min. At the respective time points, 100 µL was taken out of the well, a 1:10 serial dilution series was prepared with 900 µL anoxic 1× PBS, and 5 µL of each dilution was spotted on BHI plates for CFU determination. Plates were incubated for 3 days at 37°C before taking out the plates of the anaerobic chamber for CFU counting.

### Phylogenetic tree construction

The phylogenetic tree in Fig. 4A was built based on 16S rRNA sequences of the six Fusobacterium strains. First, the genomes were screened for 16S rRNA genes using barrnap (v0.9), and the resulting coordinates were used to extract the corresponding sequences with bedtools (v2.31.1). Multiple sequence alignment was performed with MAFFT (v7.490) using default parameters. A maximum-likelihood phylogenetic tree was then inferred from the aligned 16S rRNA sequences using FastTree (v2.1), applying its nucleotide mode and default evolutionary model. The resulting tree was then visualized with iTOL (v7.0).

### PNA treatment for RNA-seq analysis

Three biological replicates for each sample were grown overnight in ColB, diluted the following morning 1:50 in fresh ColB, and after the culture reached OD_600_ 0.5, it was diluted 1:250 to 1 × 10^6^ CFU per mL in MHB; 5 mL of the solution was transferred into 5 mL low-binding tubes (LABsolute) and incubated with 10 µM PNA or the respective volume of water for 30 min and 16 h at 37°C in the anaerobic chamber. The reaction was stopped at the mentioned time points by adding RNAprotect Bacteria (Qiagen).

### RNA isolation

To isolate fusobacterial RNA, the samples were incubated 1:1 (vol:vol) with RNAprotect Bacteria (Qiagen) for 5 min at RT, snap-frozen in liquid nitrogen, and stored at −80°C until further processing. To purify RNA, the miRNeasy Micro kit (Qiagen) was used. Briefly, cell dilutions were thawed on ice and centrifuged at 13,000 × *g* at 4°C for 20 min; the pellets resuspended in 100 µL of 0.5 mg/mL lysozyme (Roth) in TE buffer pH 8.0 and incubated for 2 min at RT. The samples were then incubated with 700 µL Qiazol (Qiagen) for 5 min at RT, and after the addition of 160 µL chloroform, the samples were shaken vigorously for 15 s to obtain phase separation. The upper aqueous phase was mixed 1:1.5 with 100% ethanol and loaded onto the Qiagen columns. Column washes, DNase I digest, and elution were performed according to the manual instructions. RNA concentrations were determined via NanoDrop measurements, and the samples were stored at −80°C until sequencing.

### RNA sequencing, quantification, and differential expression

RNA samples were processed and sequenced at the Core Unit SysMed (University of Würzburg, Germany). RNA quality was investigated using the 2100 Bioanalyzer together with the RNA 6000 Pico kit (Agilent Technologies). Ribosomal RNA (rRNA) depletion was performed using the RiboCop META rRNA depletion kit (Lexogen) according to the manufacturer’s instructions. Depleted RNA was further fragmented via ultrasound for 30 s at 4°C. After adapter ligation to the 3′ end, the first-strand cDNA was synthesized using the M-MLV reverse transcriptase. Following purification, 5′ Illumina TruSeq adapters were ligated to the cDNA, the cDNA was amplified by PCR, and the resulting product was purified with the Agencourt AMPure XP kit (Beckman Coulter Genomics). After performing library quality control using the 2100 Bioanalyzer with the DNA High Sensitivity kit (Agilent Technologies), the cDNA was pooled, purified, and sequenced using an Illumina NextSeq 500 system with 10 million reads/sample in single-end mode with 75 nt read length. RNA-seq raw reads were trimmed, filtered, and mapped using the *F. nucleatum* subsp. *nucleatum* ATCC 23726 (NZ_CP028109.1) annotation file with custom annotation, and for *F. nucleatum* subsp. *vincentii* ATCC 49256, the annotation file of *F. nucleatum* subsp. *vincentii* 3_1_36A2 (NZ_ CP003700.1) was used. To remove adapter sequences and trim nucleobases, BBDuk was used before mapping the reads with BBMap (v38.84). Differential gene expression was investigated using edgeR (v3.34.1) after assigning mapped reads to genes with the featureCounts method of the Subread (2.0.1) package. For further downstream analysis, R/Bioconductor packages were used. The reads were normalized by the trimmed mean of *M* values (TMM) normalization. Data of three biological replicates showing a log_2_ fold change of <−1.5 or >1.5 with a false discovery rate (FDR) ≤0.01 were considered differentially regulated transcripts.

### KEGG pathway enrichment analysis

Genes were assigned to KEGG pathways using the R package KEGGREST (1.32.0). To investigate the enrichment of pathways in differentially expressed transcripts, rotation gene set testing was applied. All pathways with more than 10 transcripts and an FDR-adjusted *P*-value <0.05 were visualized as statistically significant via asterisk. The color in the heat map indicates the median log_2_ FC of the respective pathway.

## Data Availability

RNA-seq data can be accessed at NCBI’s GEO (https://www.ncbi.nlm.nih.gov/geo) under the accession number GSE284320. The code used for RNA-seq data analysis is available at https://github.com/jakobjung/fuso_PNA_rnaseq.

## SUPPLEMENTAL MATERIAL

Data S1 mbio.00524-25-s0001.xlsx Overview of RNA-seq data.

Supplemental Figures and Tables mbio.00524-25-s0002.pdf Fig. S1-S6; Tables S1 and S2.

ASM does not own the copyrights to Supplemental Material that may be linked to, or accessed through, an article. The authors have granted ASM a non-exclusive, world-wide license to publish the Supplemental Material files. Please contact the corresponding author directly for reuse.
