# Supplementary material for: An antisense oligomer conjugate with unpredicted bactericidal activity against Fusobacterium nucleatum
Source: mBio. 2025 Apr 29;16(6):e00524-25. doi: 10.1128/mbio.00524-25 (PMC12153356; doi:10.1128/mbio.00524-25)
Supplement: Supplemental Figures and Tables — Fig. S1-S6; Tables S1 and S2. [file mbio.00524-25-s0002.pdf]

# An antisense oligomer conjugate with unpredicted bactericidal activity against *Fusobacterium nucleatum*

Valentina Cosi[1], Jakob Jung[1], Linda Popella[2,3], Falk Ponath[1],

Chandradhish Ghosh[1], Lars Barquist[1,4], Jörg Vogel[1-3,*]

[1] Helmholtz Institute for RNA-based Infection Research (HIRI), Helmholtz Centre for Infection Research (HZI), Würzburg, Germany

[2] RNA Biology Group, Institute for Molecular Infection Biology (IMIB), University of Würzburg, Würzburg, Germany

[3] Cluster for Nucleic Acid Therapeutics Munich (CNATM), Munich, Germany

[4] Department of Biology, University of Toronto, Mississauga, Ontario, L5L 1C6 Canada

*Correspondence: joerg.vogel@uni-wuerzburg.de

Running title: Bactericidal conjugate for fusobacteria

Keywords: *Fusobacterium nucleatum*, cell-penetrating peptide, peptide nucleic acid, morpholino, antisense antibiotic, envelope stress

This file includes:
Supplementary figures
Supplementary tables

Fig. S1

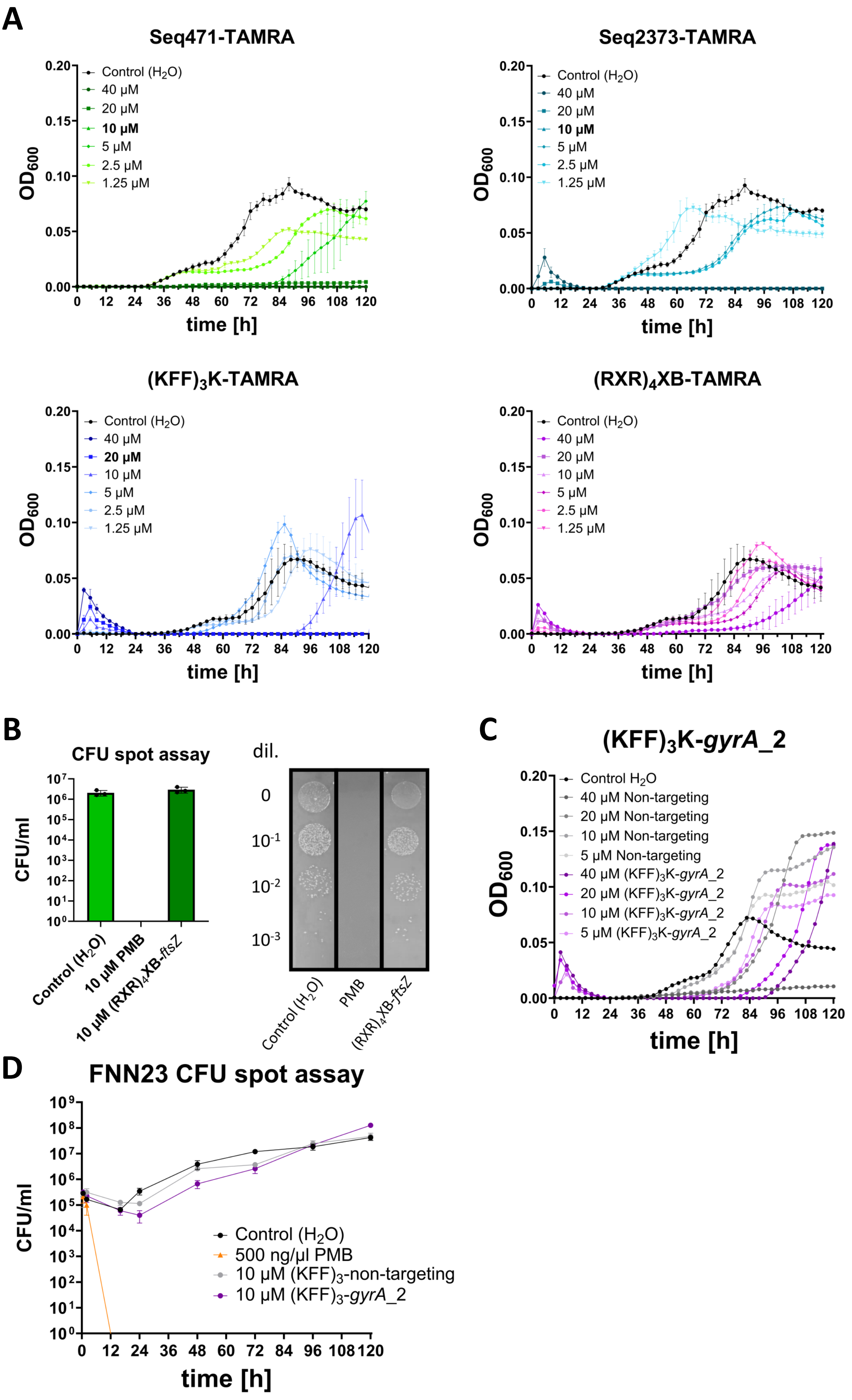
A
Seq471-TAMRA
Control (H2O)
40 µM
20 µM
10 µM
5 µM
2.5 µM
1.25 µM
OD600
time [h]
Seq2373-TAMRA
(KFF)3K-TAMRA
(RXR)4XB-TAMRA
B
CFU spot assay
CFU/ml
Control (H2O)
10 µM PMB
10 µM (RXR)4XB-ftsZ
dil.
0
10-1
10-2
10-3
PMB
(RXR)4XB-ftsZ
C
(KFF)3K-gyrA_2
Control H2O
40 µM Non-targeting
20 µM Non-targeting
10 µM Non-targeting
5 µM Non-targeting
40 µM (KFF)3K-gyrA_2
20 µM (KFF)3K-gyrA_2
10 µM (KFF)3K-gyrA_2
5 µM (KFF)3K-gyrA_2
D
FNN23 CFU spot assay
500 ng/µl PMB
10 µM (KFF)3-non-targeting
10 µM (KFF)3-gyrA_2

**Fig. S1: MIC determination of CPP-TAMRA conjugates and investigation of $(RXR)_4$XB-*ftsZ* as well as $(KFF)_3$K-*gyrA*_2.**

(A) Growth kinetics of FNN23 incubated with CPP-TAMRA conjugates Seq471, Seq2373, $(KFF)_3$K or $(RXR)_4$XB in MHB with 1 x $10^5$ CFU per ml input. Growth curves are depicted as $OD_{600nm}$ over time for three technical replicates, error bars indicate the standard deviation of three experminents. (B) Spot assay of FNN23 incubated with 10 µM $(RXR)_4$XB-*ftsZ* to test its effect on colony formation together with $H_2O$ as negative control and PMB as positive control. At 96 h post treatment, 100 µl of each sample were collected and diluted 1:10 in 1x PBS to enumerate the number of viable cells (CFU/ml) via spotting on BHI agar plates. (Left) After three days CFUs were counted for three technical replicates and quantified. Error bars represent standard deviation of three experiments. (Right) Exemplary images out of three replicates showing CFUs on BHI plates from treated samples. (C) Growth kinetics of FNN23 incubated with water, $(KFF)_3$K-*gyrA*_2 or $(KFF)_3$K-non-targeting control in MHB with 1 x $10^5$ CFU per ml input. Growth curves are depicted as $OD_{600nm}$ over time. (D) Determination of bactericidal effect kinetics for 10 µM $(KFF)_3$K-*gyrA*_2 or $(KFF)_3$K-non-targeting control as well as $H_2O$ as negative control and 10 µM PMB as positive control. At selected time points post treatment, 100 µl of each sample were collected and diluted 1:10 in 1x PBS to enumerate the number of viable cells (CFU/ml) via spotting on BHI agar plates. After three days incubation at 37°C CFUs on plates were counted and quantified for each condition. Error bars represent standard deviation of three experiments.

Fig. S2

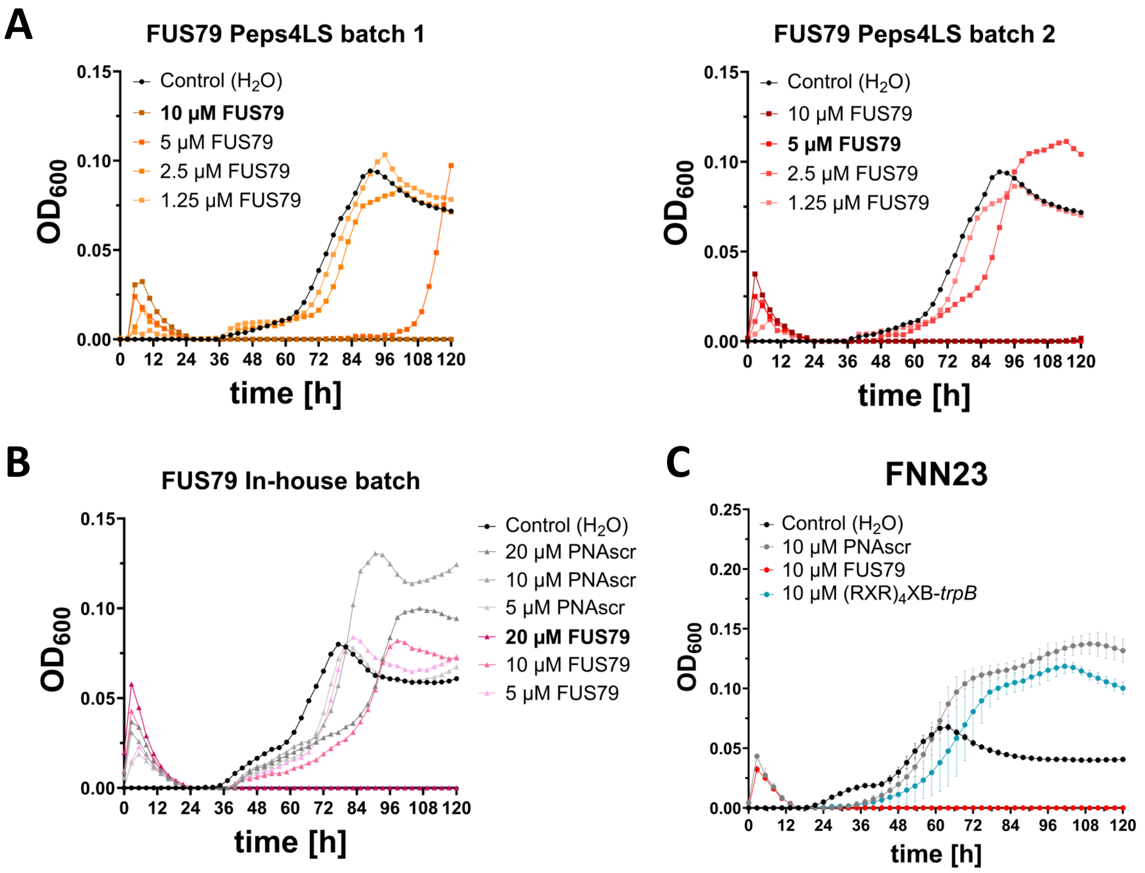


**Fig. S2: Comparison of different batches FUS79 and *trpB* off-target investigation.**

(A) Growth kinetics of FNN23 incubated with different Peps4LS batches of FUS79 or water as control. (B) Growth kinetics of FNN23 incubated with in house synthetized FUS79 or corresponding scrambled control (PNAscr), or water as control. (C) Growth kinetics of FNN23 incubated with 10 µM FUS79 (red), PNAscr (gray) or $(RXR)_4$XB-PNA complementary to TIR off-target *trpB* (teal). Error bars represent the standard deviation of three experiments. All growth curves are depicted as $OD_{600nm}$ over time with 1 x $10^5$ CFU per ml input in MHB.

Fig. S3

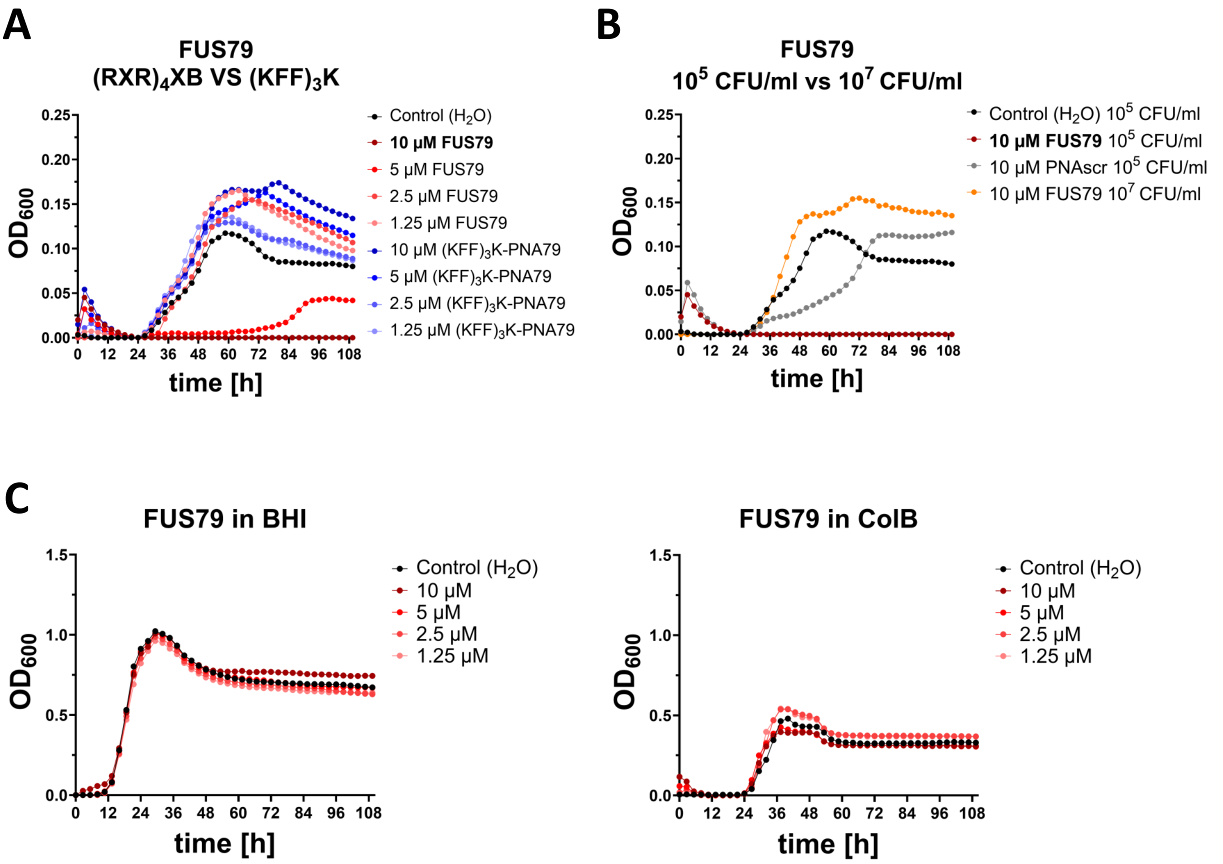


**Fig. S3: Investigation of CPP-, inoculum-, and media-dependency of the antibacterial activity of FUS79.**

(A) Growth kinetics of FNN23 incubated with FUS79 (=$(RXR)_4$XB-PNA79, red), $(KFF)_3$K-PNA79 (blue), or water control (black) in MHB with 1 x $10^5$ CFU per ml input. (B) Growth kinetics of FNN23 in MHB incubated with 10 µM FUS79 (red), PNAscr (gray) or water control (black) using 1 x $10^5$ CFU versus 10 µM FUS79 using 1 x $10^7$ CFU per ml input (orange). (C) Growth kinetics of FNN23 incubated with FUS79 (red) or water (black) in cation- and peptide-rich growth media using 1 x $10^5$ CFU per ml input: BHI (left) and ColB (right). All growth curves depicted as $OD_{600nm}$ over time.

Fig. S4

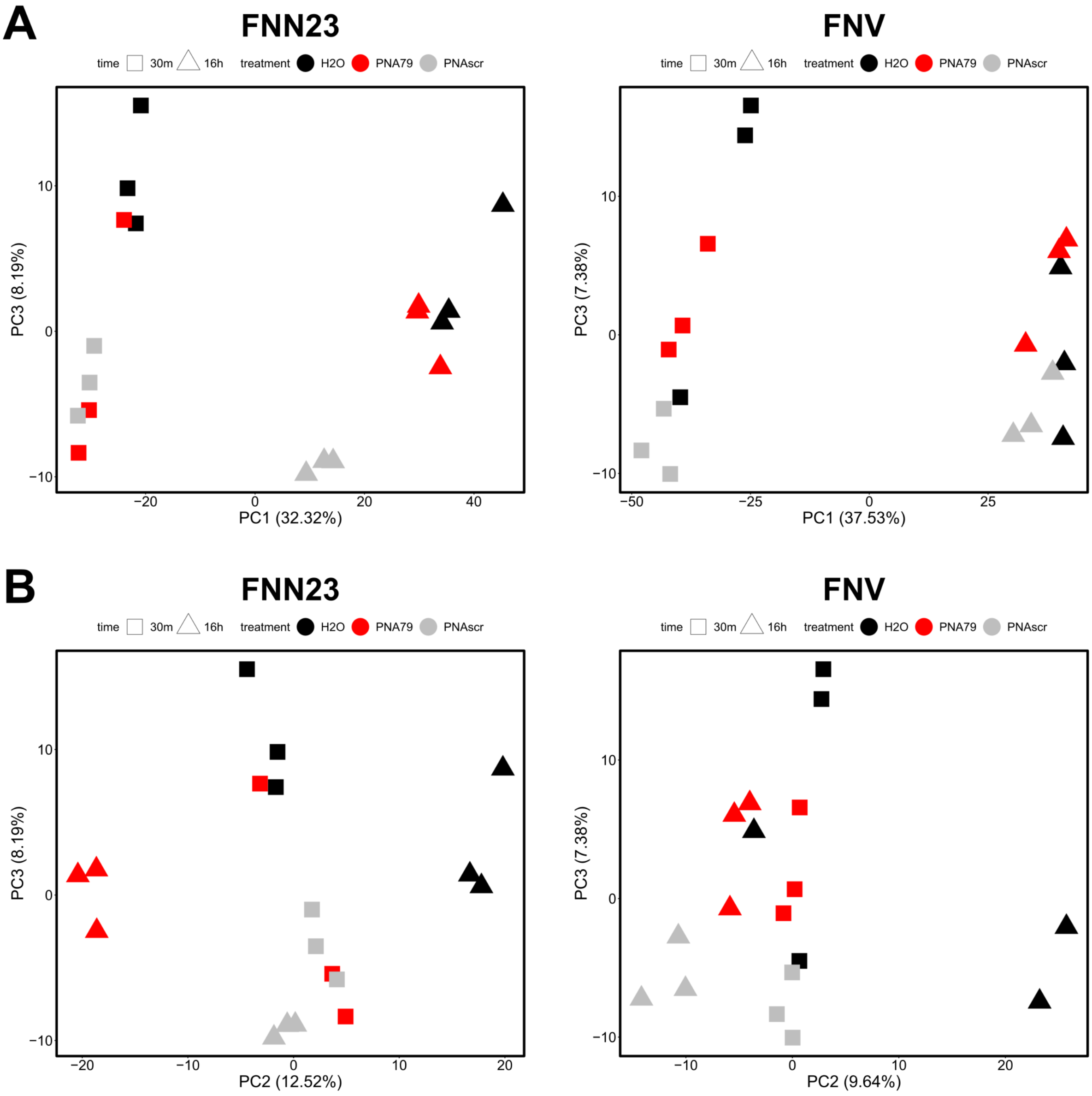


**Fig. S4: Principal component analysis of RNA-seq: PC1 vs PC3 and Pc2 vs PC3.**

Principal component analysis (PCA) of 18 samples for each fusobacterial strain after trimmed mean of M-values (TMM) normalization at 30 minutes and 16 hours after treatment. (A) PC1 vs PC3 and (B) PC2 vs PC3.

Fig. S5

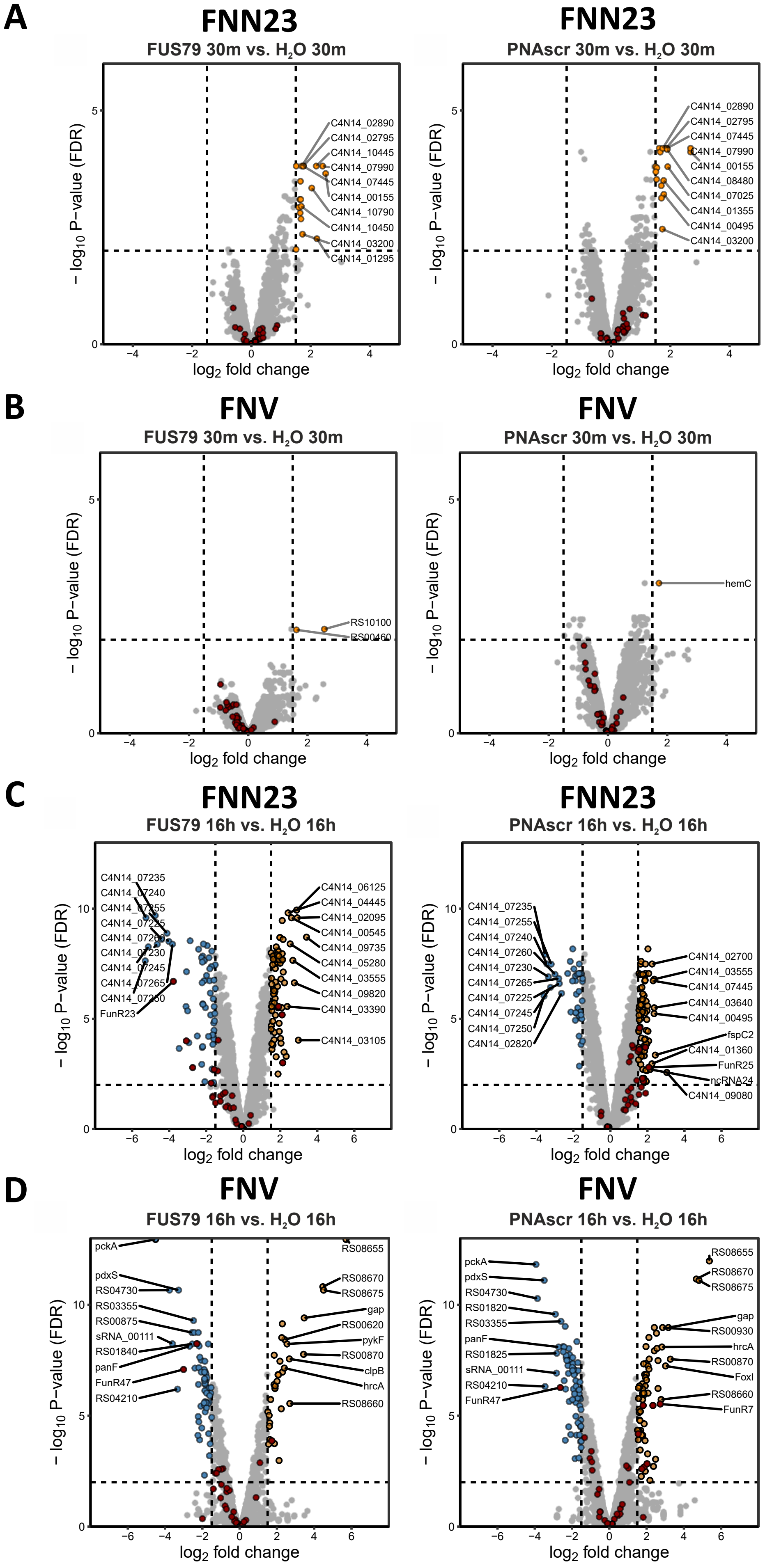

A
FNN23
FUS79 30m vs. H$_2$O 30m
C4N14_02890
C4N14_02795
C4N14_10445
C4N14_07990
C4N14_07445
C4N14_00155
C4N14_10790
C4N14_10450
C4N14_03200
C4N14_01295
FNN23
PNAscr 30m vs. H$_2$O 30m
C4N14_02890
C4N14_02795
C4N14_07445
C4N14_07990
C4N14_00155
C4N14_08480
C4N14_07025
C4N14_01355
C4N14_00495
C4N14_03200
B
FNV
FUS79 30m vs. H$_2$O 30m
RS10100
RS00460
FNV
PNAscr 30m vs. H$_2$O 30m
hemC
C
FNN23
FUS79 16h vs. H$_2$O 16h
C4N14_07235
C4N14_07240
C4N14_07255
C4N14_07225
C4N14_07260
C4N14_07230
C4N14_07245
C4N14_07265
C4N14_07250
FunR23
C4N14_06125
C4N14_04445
C4N14_02095
C4N14_00545
C4N14_09735
C4N14_05280
C4N14_03555
C4N14_09820
C4N14_03390
C4N14_03105
FNN23
PNAscr 16h vs. H$_2$O 16h
C4N14_07235
C4N14_07255
C4N14_07240
C4N14_07260
C4N14_07230
C4N14_07265
C4N14_07225
C4N14_07245
C4N14_07250
C4N14_02820
C4N14_02700
C4N14_03555
C4N14_07445
C4N14_03640
C4N14_00495
fspC2
C4N14_01360
FunR25
ncRNA24
C4N14_09080
D
FNV
FUS79 16h vs. H$_2$O 16h
pckA
pdxS
RS04730
RS03355
RS00875
sRNA_00111
RS01840
panF
FunR47
RS04210
RS08655
RS08670
RS08675
gap
RS00620
pykF
RS00870
clpB
hrcA
RS08660
FNV
PNAscr 16h vs. H$_2$O 16h
pckA
pdxS
RS04730
RS01820
RS03355
panF
RS01825
sRNA_00111
RS04210
FunR47
RS08655
RS08670
RS08675
gap
RS00930
hrcA
RS00870
FoxI
RS08660
FunR7
− log$_{10}$ P−value (FDR)
log$_2$ fold change

**Fig. S5: Transcriptomic profiling of FNN23 and FNV in response to $(RXR)_4$XB-PNA treatment.**

Transcriptomic response of FNN23 (A) and FNV (B) upon PNA-FUS79 or respective PNAscr treatment compared to $H_2O$ control at 30 minutes and transcriptomic response of FNN23 (C) and FNV (D) upon PNA-FUS79 or PNAscr treatment compared to $H_2O$ control at 16 hours. For A-D volcano plots show differential gene expression as $-\log_{10}$ false discovery rate (FDR)-adjusted P-values on y-axis and $\log_2$ fold change on x-axis. Significantly differentially expressed transcripts are defined by an absolute fold change <-1.5 or >1.5 and an FDR adjusted P-value <0.01, characterized by the dashed lines in the plot. Significantly upregulated transcripts are depicted in orange, significantly downregulated transcripts in blue and all sRNAs dots are colored in red. The top ten differentially expressed transcripts are specified by the depicted locus tag.

Fig. S6

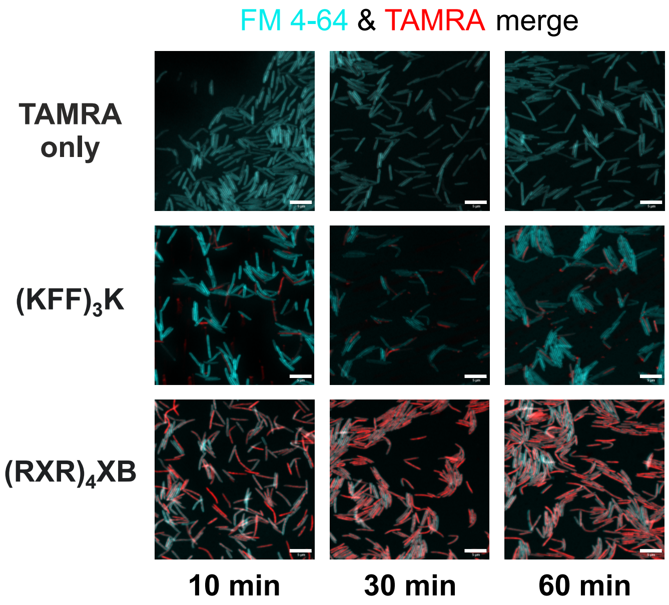


**Fig. S6: Investigation of CPP-TAMRA penetration in FNV using CLSM.**

Representative CLSM images for TAMRA only control or TAMRA-labeled CPPs (red) at 5 μM and 10, 30 and 60 minutes post incubation. FNV was incubated with FM 4-64 (cyan) to stain the cell membrane, shown in cyan. Scale bar, 5 μm.

## Supplementary Tables

*Table S1: Cell penetrating peptides*

| | Name | Peptide sequence (N to C terminus) | Modification (N-terminus) | Source |
|---|---|---|---|---|
| 1 | $(KFF)_3K$ | KFFKFFKFFK | - | Peps4LS |
| 2 | $(KFF)_3K$ | KFFKFFKFFK | 5,6-TAMRA | Peps4LS |
| 3 | $(RXR)_4XB$ | RXRRXRRXRRXRXB* | - | Peps4LS |
| 4 | $(RXR)_4XB$ | RXRRXRRXRRXRXB* | 5,6-TAMRA | Peps4LS |
| 5 | Seq471 | WLRRIKAWLRRIKALNRQLGVAA | 5,6-TAMRA | Peps4LS |
| 6 | Seq2373 | AHKLKKPKIVRLIKFLLKAWK | 5,6-TAMRA | Peps4LS |

*X is 6-aminohexanoic acid; B is β-alanine.

*Table S2: Antisense oligomer conjugates*

| | Name | Target | Peptide sequence† | ASO sequence# | Length | Source |
|---|---|---|---|---|---|---|
| 1 | $(KFF)_3K$-*acpP* | *acpP* | KFFKFFKFFK | ACATATTCTC | 10mer | Peps4LS |
| 2 | $(KFF)_3K$-*acpP*-scr | - | KFFKFFKFFK | TACATCTCTT | 10mer | Peps4LS |
| 3 | $(RXR)_4XB$-*acpP* | *acpP* | RXRRXRRXRRXRXB | ACATATTCTC | 10mer | Peps4LS |
| 4 | $(RXR)_4XB$-*acpP*-scr | - | RXRRXRRXRRXRXB | TACATCTCTT | 10mer | Peps4LS |
| 5 | $(KFF)_3K$-*ftsZ* | *ftsZ* | KFFKFFKFFK | TGTCATAAAT | 10mer | Peps4LS |
| 6 | $(KFF)_3K$-*ftsZ*-scr | - | KFFKFFKFFK | ACGATATTTA | 10mer | Peps4LS |
| 7 | $(RXR)_4XB$-*ftsZ* | *ftsZ* | RXRRXRRXRRXRXB | TGTCATAAAT | 10mer | Peps4LS |
| 8 | $(RXR)_4XB$-*ftsZ*-scr | - | RXRRXRRXRRXRXB | ACGATATTTA | 10mer | Peps4LS |
| 9 | $(KFF)_3K$-*gyrA* | *gyrA* | KFFKFFKFFK | TTGACATTTA | 10mer | Peps4LS |
| 10 | $(KFF)_3K$-*gyrA*-scr | - | KFFKFFKFFK | GCTTATATAT | 10mer | Peps4LS |
| 11 | $(RXR)_4XB$-*gyrA* | *gyrA* | RXRRXRRXRRXRXB | TTGACATTTA | 10mer | Peps4LS |
| 12 | $(RXR)_4XB$-*gyrA*-scr | - | RXRRXRRXRRXRXB | GCTTATATAT | 10mer | Peps4LS |
| 13 | $(KFF)_3K$-*acpP_11mer* | *acpP* | KFFKFFKFFK | ACATATTCTCC | 11mer | Peps4LS |
| 14 | $(KFF)_3K$-*acpP_15mer* | *acpP* | KFFKFFKFFK | ACATATTCTCCTCCT | 15mer | Peps4LS |
| 15 | D-$(KFF)_3K$-*acpP* | *acpP* | $K^DFFK^DFFK^DFFK^D$ | ACATATTCTC | 10mer | Peps4LS |
| 16 | D-$(KFF)_3K$-*non-targeting_10mer* | - | $K^DFFK^DFFK^DFFK^D$ | CGCATTGCGA | 10mer | Peps4LS |
| 17 | $(KFF)_3K$-*non-targeting_11mer* | - | KFFKFFKFFK | CGCATTGCGAA | 11mer | Peps4LS |
| 18 | $(KFF)_3K$-*non-targeting_15mer* | - | KFFKFFKFFK | CTTTTGCCATAGTCT | 15mer | Peps4LS |
| 19 | $(KFF)_3K$-*dnaB* | *dnaB* | KFFKFFKFFK | TTTGACATTTA | 11mer | Peps4LS |

| | | | | | | |
|---|---|---|---|---|---|---|
| 20 | (KFF)$_3$K-*clpB* | *clpB* | KFFKFFKFFK | GGATTCATCAT | 11mer | Peps4LS |
| 21 | (KFF)$_3$K-*gyrA_11mer* | *gyrA* | KFFKFFKFFK | TTTGACATTTA | 11mer | Peps4LS |
| 22 | (KFF)$_3$K-*gyrA_11mer_2* | *gyrA* | KFFKFFKFFK | CATTTGACATT | 11mer | Peps4LS |
| 23 | (KFF)$_3$K-*groEL* | *groEL* | KFFKFFKFFK | TAACCTCCTA | 10mer | Peps4LS |
| 24 | (KFF)$_3$K-*rpoA* | *rpoA* | KFFKFFKFFK | AACATTCTATC | 11mer | Peps4LS |
| 25 | (KFF)$_3$K-*rpoB* | *rpoB* | KFFKFFKFFK | GCACGTTTCAC | 11mer | Peps4LS |
| 26 | (KFF)$_3$K-*rpoD* | *rpoD* | KFFKFFKFFK | GCTCTTTCAC | 10mer | Peps4LS |
| 27 | (RXR)$_4$XB-*acpP_2* | *acpP* | RXRRXRRXRRXRXB | AACATATTCT | 10mer | Peps4LS |
| 28 | (RXR)$_4$XB-*ftsZ_2* | *ftsZ* | RXRRXRRXRRXRXB | TCTGTCATAA | 10mer | Peps4LS |
| 29 | (RXR)$_4$XB-*gyrA_2* | *gyrA* | RXRRXRRXRRXRXB | CATTTGACAT | 10mer | Peps4LS |
| 30 | (RXR)$_4$XB-*fomA* | *fomA* | RXRRXRRXRRXRXB | GGTTTTTTCCC | 11mer | Peps4LS |
| 31 | (RXR)$_4$XB-non-targeting_10mer | - | RXRRXRRXRRXRXB | ATCGATTTAC | 10mer | Peps4LS |
| 32 | (RXR)$_4$XB-non-targeting_11mer (=FUS79) | - | RXRRXRRXRRXRXB | GACATAATTGT | 11mer | Peps4LS |
| 33 | PNAscr | - | RXRRXRRXRRXRXB | AGTCATTAATG* | 11mer | Peps4LS |
| 34 | FUS79 variant 1 | - | RXRRXRRXRRXRXB | GACATATTAGT* | 11mer | Peps4LS |
| 35 | PNA79 | - | -* | GACATAATTGT | 11mer | Peps4LS |
| 36 | (RXR)$_3$XB-PNA79 | - | RXRRXRRXRXB* | GACATAATTGT | 11mer | Peps4LS |
| 37 | FUS79 variant 2 | - | RXRRXRRXRRXRXB | GCAATAATGTT* | 11mer | Peps4LS |
| 38 | (RXR)$_4$XB-*trpB* | *trpB* | RXRRXRRXRRXRXB | GCCATAATTTT* | 11mer | Peps4LS |
| 39 | (RXR)$_4$XB-FUS79_HZI | - | RXRRXRRXRRXRXB | GACATAATTGT | 11mer | HZI |
| 40 | (RXR)$_4$XB-non-targeting_HZI | - | RXRRXRRXRRXRXB | ATACCTTTACG | 11mer | HZI |
| 41 | (KFF)$_3$K-non-targeting-PMO | - | KFFKFFKFFK | TCACTATCTCTGT | 13mer | Gene Tools |
| 42 | (KFF)$_3$K-PMO79 | - | KFFKFFKFFK | GACATAATTGT | 11mer | Gene Tools |
| 43 | (RXR)$_4$XB-non-targeting-PMO | - | RXRRXRRXRRXRXB | CACTATCTCTG | 11mer | Gene Tools |
| 44 | (RXR)$_4$XB-PMO79 | - | RXRRXRRXRRXRXB | GACATAATTGT | 11mer | Gene Tools |

[†]Peptide sequences are shown from N to C terminus. [#]PNA sequences are shown from N to C terminus, PMO sequences are shown from 5’ to 3’ end. *Changes of FUS79 variants respective to the original FUS79 conjugate are highlighted in red.
